# Supplementary material for: Real-world ethics in palliative care: protocol for a systematic review of the ethical challenges reported by specialist palliative care practitioners in their clinical practice
Source: BMJ Open. 2019 May 27;9(5):e028480. doi: 10.1136/bmjopen-2018-028480 (PMC6538058; doi:10.1136/bmjopen-2018-028480)
Supplement: Supplementary data [file bmjopen-2018-028480supp001.pdf]

Medline Search Strategy

| | |
|---|---|
| 1 | Ethics/ |
| 2 | Ethics, Nursing/ |
| 3 | Ethics, Medical/ |
| 4 | Ethics, Clinical/ |
| 5 | exp Ethics, Professional/ |
| 6 | BIOETHICS/ |
| 7 | moral*.tw. |
| 8 | ethic*.tw. |
| 9 | 1 or 2 or 3 or 4 or 5 or 6 or 7 or 8 |
| 10 | Palliative Care/ |
| 11 | Palliative Medicine/ |
| 12 | Terminal Care/ |
| 13 | Hospice Care/ |
| 14 | Hospices/ |
| 15 | ((end of life or terminal*) adj3 (ill* or care)).tw. |
| 16 | palliat*.tw. |
| 17 | hospice*.tw. |
| 18 | 10 or 11 or 12 or 13 or 14 or 15 or 16 or 17 |
| 19 | 9 and 18 |
| 20 | exp animals/ not humans/ |
| 21 | exp Animals, Laboratory/ |
| 22 | exp Animal Experimentation/ |
| 23 | exp Models, Animal/ |
| 24 | (rat or rats or mouse or mice or rodent*).ti. |
| 25 | 20 or 21 or 22 or 23 or 24 |
| 26 | 19 not 25 |
| 27 | exp "Surveys and Questionnaires"/ |
| 28 | survey*.mp. |
| 29 | question*.mp. |
| 30 | or/27-29 |
| 31 | ((("semi-structured" or semistructured or unstructured or informal or "in-depth" or indepth or "face-to-face" or structured or guide) adj3 (interview* or discussion* or questionnaire*)) or (focus group* or qualitative or ethnograph* or fieldwork or "field work" or "key informant")).ti,ab. or interviews as topic/ or focus groups/ or narration/ or qualitative research/ |
| 32 | 30 or 31 |
| 33 | 26 and 32 |
