# Supplementary material for: Real-world ethics in palliative care: protocol for a systematic review of the ethical challenges reported by specialist palliative care practitioners in their clinical practice
Source: BMJ Open. 2019 May 27;9(5):e028480. doi: 10.1136/bmjopen-2018-028480 (PMC6538058; doi:10.1136/bmjopen-2018-028480)
Supplement: Author's manuscript [file bmjopen-2018-028480.draft_revisions.pdf]

BMJ Open

BMJ Open is committed to open peer review. As part of this commitment we make the peer review history of every article we publish publicly available.

When an article is published we post the peer reviewers' comments and the authors' responses online. We also post the versions of the paper that were used during peer review. These are the versions that the peer review comments apply to.

The versions of the paper that follow are the versions that were submitted during the peer review process. They are not the versions of record or the final published versions. They should not be cited or distributed as the published version of this manuscript.

BMJ Open is an open access journal and the full, final, typeset and author-corrected version of record of the manuscript is available on our site with no access controls, subscription charges or pay-per-view fees (http://bmjopen.bmj.com).

If you have any questions on BMJ Open's open peer review process please email info.bmjopen@bmj.com

BMJ Open

Day-to-Day Ethics in Palliative Care: Protocol for a Systematic Review of the Ethical Challenges Reported by Specialist Palliative Care Practitioners in their Clinical Practice

Journal:	*BMJ Open*
Manuscript ID	bmjopen-2018-028480
Article Type:	Protocol
Date Submitted by the Author:	10-Dec-2018

Complete List of Authors:	Schofield, Guy; University of Bristol, Centre for Ethics in Medicine<br>Brangan, Emer; University Hospitals Bristol NHS Foundation Trust, The National Institute for Health Research Collaboration for Leadership in Applied Health Research and Care West (NIHR CLAHRC West)<br>Dittborn, Mariana; King's College London, Florence Nightingale Faculty of Nursing, Midwifery & Palliative Care<br>Huxtable, Richard; University of Bristol, School of Social and Community Medicine<br>Selman, Lucy; University of Bristol School of Social and Community Medicine

Keywords:	PALLIATIVE CARE, Empirical Ethics, MEDICAL ETHICS, Systematic Review, Ethical Challenges

SCHOLARONE™ Manuscripts

Day-to-Day Ethics in Palliative Care: Protocol for a Systematic Review of the Ethical Challenges Reported by Specialist Palliative Care Practitioners in their Clinical Practice

Guy Schofield[1*], Emer Brangan[2], Mariana Dittborn[3], Richard Huxtable[4], Lucy Ellen Selman[5]

[1] Centre for Ethics in Medicine, Population Health Sciences, Bristol Medical School, University of Bristol, Canynge Hall, 39 Whatley Road, Bristol, BS8 2PS, United Kingdom Tel: +44(0)117 33 14550. Orcid ID: 0000-0002-9055-292X [* Corresponding Author]

[2] The National Institute for Health Research Collaboration for Leadership in Applied Health Research and Care West (NIHR CLAHRC West) at University Hospitals Bristol NHS Foundation Trust, Bristol, UK

[3] MSc Student, Florence Nightingale Faculty of Nursing, Midwifery & Palliative Care, King's College London, London, UK

[4] Centre for Ethics in Medicine, Population Health Sciences, Medical School, University of Bristol, Bristol, UK

[5] Population Health Sciences, Medical School, University of Bristol, Bristol, UK

Current word count: 2426

Keywords

Palliative Care, Empirical Ethics, Medical Ethics, Systematic Review, Ethical Challenges

For peer review only

Abstract

Introduction

Ethical issues arise daily in the delivery of palliative care. Despite much (largely theoretical) literature, evidence from specialist palliative care practitioners (SPCPs) about day-to-day ethical challenges has not previously been synthesised. This evidence is crucial to inform education and training and adequately support staff. The aim of this systematic review is to synthesise the evidence regarding the ethical challenges which SPCPs encounter during clinical practice.

Methods and Analysis

We will conduct a systematic review with narrative synthesis of empirical studies that use inductive methods to describe the ethical challenges reported by SPCPs. We will search multiple databases (MEDLINE, Philosopher's Index, EMBASE, PsycINFO, LILACS, WHOLIS, Web of Science and CINAHL) without time, language or geographical restrictions. Keywords will be developed from scoping searches, consultation with information specialists, and reference to key systematic reviews in palliative care and bioethics. Reference lists of included studies will be hand-searched. 10% of retrieved titles and abstracts will be independently dual screened, as will all full text papers. Quality will be dual assessed using the Mixed-Methods Appraisal Tool (2011). Narrative synthesis following Popay et al (2006) will be used to synthesise findings. The strength of resulting recommendations will be assessed using the Grading of Recommendations Assessment, Development and Evaluation approach for qualitative evidence (GRADE-CERQual)

Ethics and Dissemination

As this review will include only published data, no specific ethical approval is required.

We anticipate that the systematic review will be of interest to palliative care practitioners of all backgrounds, and educators in palliative care and medical ethics. Findings will be presented at conferences and published open access in a peer-reviewed journal.

PROSPERO Registration number: CRD42018105365

For peer review only

Strengths and Limitations

i) The systematic review search strategy utilises a broad range of electronic databases, including those which index philosophical as well as clinical research and international publications.

ii) This global review benefits from no language, time or location restrictions in the search strategy.

iii) The use of peer-reviewed filters for qualitative and survey-based methodologies may lead to loss of some relevant studies.

iv) The exclusion of non-inductive studies investigating single ethical issues, such as palliative sedation, risks reducing the depth of detail that will be incorporated into the final synthesis.

v) However, the benefit of including only inductive studies is that the resultant synthesis will represent only those topics that are directly reported by SPCPs, reflecting the real-world context.

For peer review only

For peer review only - http://bmjopen.bmj.com/site/about/guidelines.xhtml

INTRODUCTION

Palliative care is a holistic approach to the care of patients with life-limiting illness that aims to maximise quality of life.[1] The focus of care includes both the patient and those close and important to them, such as their family.

Despite the increasing global provision of palliative care services, the need for palliative care is growing and unmet.[2,3] In 2011, 74% of countries worldwide had either no, or only isolated palliative care services.[4] The 2017 Lancet Commission Report estimated that globally 61.1m people required specialist palliative care input in 2015.[5] The majority of these people live in Low and Middle Income Countries, where provision of specialist palliative care is highly variable; globally, it is estimated that only 14% of those who might benefit from palliative care receive it.[2] In the United Kingdom (UK), modelling predicts that by 2040 there will be both an increase in the absolute numbers of deaths, and, due to multimorbidity and medical complexity, an increase in the percentage of those dying that require specialist palliative care.[6] As the current worldwide epidemic of non-communicable diseases grows, this trend is likely to be replicated.[7,8]

Palliative care is frequently connected with moral problems across aspects of clinical care.[9] These include, for example, withdrawing and withholding of interventions,[10] dignity and quality of care,[11] respect for autonomy[12] and palliative sedation.[13,14] Unusually for a healthcare field, the most commonly used definition, that of the World Health Organization, is explicitly value-laden in calling for the 'impeccable' assessment and treatment of pain and other problems.[1] The UK Palliative Medicine curriculum from the Royal College of Physicians contains detailed content related to ethics-based competencies,[15] and the need for training in the ethical aspects of the field is recognised as a priority.[16]

In the field of bioethics there has recently been an 'empirical turn', central to which is the idea that understanding the real-world context of moral problems is a key part of their analysis.[17] Fundamental to high-quality empirical bioethics is an accurate understanding of context, taking robust empirical evidence as a starting point.[18] Education, too, benefits from a robust grounding in the real-world experiences of learners: the relevance of educational material is a key factor in adult learner motivation,[19] and processing new material in relation to prior experiences contributes to learning efficiency.[20] In ethics training, a thorough understanding of the ethical context practitioners work within is needed if educators are to generate evidence-based curricula that reflect real world contexts.

For peer review only - http://bmjopen.bmj.com/site/about/guidelines.xhtml

But despite its potential benefits to both clinical ethics and ethics education, empirical evidence of the ethical challenges faced by specialist palliative care providers (SPCPs) is rarely referenced in these fields. Furthermore, there is evidence from other areas of healthcare practice that the ethical dilemmas that are written about in the literature do not reflect the range of the dilemmas that healthcare workers report experiencing on a day-to-day basis.[21–23] While this has not previously been systematically examined within palliative care, there is some evidence suggesting this mismatch also applies.[24–26] Hermsen and Ten Have,[24] for example, compared the ethical challenges reported by SPCPs from the Netherlands with those found in the palliative care literature. They found 14 reported ethical challenges with no accompanying literature, and two topics with significant literature (organ donation and engagement with ethical committees), but which were not reported in practice.[24]

There is a need, therefore, to systematically review and synthesise the published evidence regarding the ethical challenges reported by SPCPs.

Aim

We aim to systematically review the literature to answer the following research question: what do specialist palliative care practitioners report as ethical challenges that they experience during clinical practice?

METHODS AND ANALYSIS

Eligibility Criteria

This review aims to identify studies that describe the ethical challenges reported by SPCPs in their day-to-day clinical practice. The inclusion and exclusion criteria are summarised in Table 1. Butler et al. describe an adaptation of the population, intervention, comparison, and outcome (PICO) system for systematic reviews that are likely to be processing qualitative research[27]; we use their Population, Context and Outcome (PCO) system.

Table 1. Inclusion and exclusion criteria

	Inclusion criteria	Exclusion criteria
Types of participants	Study participants are specialist palliative care practitioners (SPCPs). We define SPCPs as people working in, or for, a healthcare setting whose main focus is on delivering palliative care (as opposed to clinical contexts where palliative care forms part, but not the main focus, of the care provided).	Participants who undertake palliative care tasks as part of their role (e.g. oncologists), but who do not specialise in providing palliative care and do not have palliative care as the main focus of their role.

For peer review only - http://bmjopen.bmj.com/site/about/guidelines.xhtml

	This may include (but is not limited to) nurses, doctors, occupational therapist, physiotherapists, dieticians, speech and language therapists, psychologists, other allied health professionals and chaplains.<br><br>Studies with a mixed population where SPCP participants' data are separately presented and can be extracted will be included.	
Context	All geographical settings and all clinical settings where specialist palliative care (SPC) is delivered will be included.	Studies conducted in settings in which SPC is not being delivered.
Types of outcomes	Ethical challenges that are reported as experienced by SPCPs during clinical delivery of palliative care.<br><br>The definition of 'ethical challenges' will be intentionally kept broad to capture the maximum number of examples. It includes but is not limited to terms such as ethical issues, moral challenges, moral dilemmas, values, good/bad, right/wrong. Ethical challenges can be labelled as such either by authors or participants.	Studies that utilise survey tools with pre-selected ethical dilemmas that have not been inductively derived based on evidence from SPCPs, and studies that investigate a single aspect of palliative care only will be excluded.<br><br>These study designs are excluded as they proceed from an a priori assumption that their selected issues are relevant. They therefore do not contribute to an inductive exploration of the breadth and type of ethical challenges facing practitioners.
Type of studies	Empirical qualitative or quantitative studies examining ethical challenges reported as being experienced by SPCPs. Survey studies reporting free text data will be included.	Studies not reporting inductive empirical data.
Timeframe	Any time frame up until the search date will be included, contingent on the inception dates of the databases included in the search.	
Type of publications	Peer-reviewed journal publications of empirical research. Papers in any language will be included, with findings translated into English.	Where no full text is available through the university subscription, study authors will be contacted for full text. If there is no response within two weeks the study will be excluded.<br>The following will also be excluded:<br>- Conference abstracts; however, authors will be contacted for further data/publications.<br>- Editorials, letters, or comment/opinion pieces.<br>- Review articles. Reviews will be used for identification of primary research only.<br>- Book sections.

SPCP = specialist palliative care provider

The review will include peer-reviewed inductive studies with primary data derived from SPCPs reporting the ethical challenges they face in day-to-day practice, or secondary analyses of such data. Inductive research aims to report the experience of the participants, and may also derive theory

For peer review only - http://bmjopen.bmj.com/site/about/guidelines.xhtml

from this data. Inductive data collection occurs independently from any attempt to validate a particular theory, test a defined hypothesis, or explore a pre-selected topic.

Non-peer reviewed papers, studies not reporting inductive empirical data, book chapters, editorials and theses, case reports, opinion pieces, and reviews will be excluded.

We will include only inductive studies as we aim to generate a landscape of experienced challenges from the real-world context. Scoping searches identified multiple studies investigating pre-selected ethical challenges within the practice of palliative care. Studies of this type will be excluded as they reflect choices of the study authors rather than the real-world experience of practitioners; including them in the synthesis would risk introducing data that do not reflect SPCPs experiences.

There will be no language or timeframe restrictions.

Search Strategy

Electronic Searches

The following databases, identified in conjunction with subject information specialists and indexing journals containing key papers known to the research team, will be searched: MEDLINE (Ovid interface, 1946 onwards), Philosopher's Index (OVID interface 1940 onwards), EMBASE (OVID interface, 1980 onwards), PsycINFO (OVID interface 1806 onwards), LILACS (http://lilacs.bvsalud.org/en/ 1982 onwards), Web of Science (Clarivate interface, 1900 onwards) and CINAHL (EBSCO interface, 1937 onwards). There will be no language, geographical or time limits.

Initial search terms were developed with reference to the key words of major systematic reviews in palliative care and bioethics. Scoping searches suggested that the initial search terms would result in over 20,000 records returned, and that relevant studies would be qualitative (e.g. using interviews or focus groups) or use survey-based methodologies to collect free-text data. To increase the specificity of the search, we will therefore apply peer-reviewed methodological filters for these study designs, identified via the InterTASC Information Specialists' Sub-Group Search Filter Resource.[28] The MEDLINE search strategy (Box 1) will be checked and modified for the other databases as appropriate.

For peer review only - http://bmjopen.bmj.com/site/about/guidelines.xhtml

Box 1. Medline Search Strategy

Medline Search Strategy

1	Ethics/
2	Ethics, Nursing/
3	Ethics, Medical/
4	Ethics, Clinical/
5	exp Ethics, Professional/
6	BIOETHICS/
7	moral*.tw.
8	ethic*.tw.
9	1 or 2 or 3 or 4 or 5 or 6 or 7 or 8
10	Palliative Care/
11	Palliative Medicine/
12	Terminal Care/
13	Hospice Care/
14	Hospices/
15	((end of life or terminal*) adj3 (ill* or care)).tw.
16	palliat*.tw.
17	hospice*.tw.
18	10 or 11 or 12 or 13 or 14 or 15 or 16 or 17
19	9 and 18
20	exp animals/ not humans/
21	exp Animals, Laboratory/
22	exp Animal Experimentation/
23	exp Models, Animal/
24	(rat or rats or mouse or mice or rodent*).ti.
25	20 or 21 or 22 or 23 or 24
26	19 not 25
27	exp "Surveys and Questionnaires"/
28	survey*.mp.
29	question*.mp.
30	or/27-29
	((("semi-structured" or semistructured or unstructured or informal or "in-depth" or i[illegible] "face-to-face" or structured or guide) adj3 (interview* or discussion* or questionnai[illegible] group* or qualitative or ethnograph* or fieldwork or "field work" or "key informant" [illegible]
31	interviews as topic/ or focus groups/ or narration/ or qualitative research/
32	30 or 31
33	26 and 32

For peer review only - http://bmjopen.bmj.com/site/about/guidelines.xhtml

Searching Other Resources

Reference lists of included papers will be hand-searched. Corresponding authors of papers meeting the inclusion criteria will be contacted to ascertain if there are other published papers they recommend for review. Authors of conference abstracts will be contacted for peer-reviewed data or follow-up publications if available; both will be included if provided and eligible. Papers that cite the included studies will be screened for inclusion.

A grey literature search will not be conducted. Cook et al. demonstrated that an extensive grey literature search did not benefit the review content of a palliative care systematic review despite the significant resources required to undertake it.[29]

Selection Process

All titles and/or abstracts of retrieved records will be screened to identify papers that potentially meet the inclusion criteria. The first researcher (GS) will screen the full search results. A second researcher (MD) will independently screen a random sample of 10%. Differences in screening between GS and the second reviewer will be discussed with the research team to clarify and refine inclusion/exclusion criteria. Contested papers will be discussed and any that remain unresolved will be examined by third reviewer (LS).

The full text of potentially eligible records will be retrieved and independently assessed for eligibility by two review team members (GS, MD). Any disagreement between them over the eligibility of particular papers will be resolved through discussion with a third reviewer (LS).

Data extraction & management

Search results will be exported and collated in Endnote X8. Records will be de-duplicated and numerical results will be recorded and presented in a flowchart that follows the PRISMA design.[30]

Data extraction will be undertaken independently by two reviewers, using a pre-piloted data extraction form. Disagreements will be resolved through consultation with a third reviewer if necessary. Data items to be extracted from included studies will include: 1) citation details including title, publication year and journal; 2) study setting, methods, participant characteristics, sample size; 3) specified definition/conceptualisation of ethical challenges; 4) key findings, themes and sub-themes; 5) sources of potential bias including funders and evidence of reflexivity. In the event of relevant missing data, corresponding authors will be contacted.

Data Synthesis

For peer review only - http://bmjopen.bmj.com/site/about/guidelines.xhtml

We will undertake a systematic narrative synthesis, following the framework stages proposed by Popay et al,[31] adapted for a review which does not focus on an intervention: (1) Developing a preliminary synthesis of study findings, (2) Exploring relationships in the data, (3) Assessing the robustness of the synthesis product. Stage 1 will include integrating the themes and content of qualitative studies; this will be guided by the 'thematic synthesis' approach developed by Thomas and Harden. [32] The narrative synthesis will explore findings within and across included studies, taking into account study quality (see below); identify patterns in the data; and synthesise the described ethical challenges in an overarching framework or model.

Risk of bias (quality) assessment

Scoping searches suggest that multiple study designs may be returned. So that the quality of diverse study designs can be compared, we will use the Mixed-Methods Assessment Tool (MMAT) (2011 Version)[33] which allows for comparison of quality between studies using differing methodologies. We will not use low MMAT scores to exclude studies, but we will reflect on study quality and the effect of lower scoring studies on the resulting synthesis. Two reviewers (GS, MD) will score each of the included studies independently. Any disagreements will be resolved by consulting a third independent reviewer.

While this review is not designed to produce recommendations for clinical practice, it is nevertheless important that we reflect on our confidence in the evidence synthesis. As the focus of the review is on inductively-derived empirical data we will use the GRADE-CERQual framework to do so.[34] CERQual provides a systematic and transparent framework for assessing confidence in individual review findings, based on consideration of four components: (1) methodological limitations, (2) coherence, (3) adequacy of data, and (4) relevance. Assessments of the four components collectively contribute to an overall assessment of whether findings from a qualitative evidence synthesis provide a reasonable representation of the phenomenon of interest.

Ethics and Dissemination

As this review will include only published data, no specific ethical approval is required.

This systematic review will synthesise empirical evidence on the ethical challenges reported by SPCPs. The research team anticipate that it will be of interest to palliative care practitioners of all backgrounds, and educators involved in palliative care or postgraduate ethics training. Findings will be presented at relevant conferences and published in a peer-reviewed journal in open access format.

Patient and public involvement

For peer review only - http://bmjopen.bmj.com/site/about/guidelines.xhtml

Patients and the public were not involved in designing the protocol of this systematic review.

DISCUSSION

Ethical challenges are a significant part of the day-to-day experience of working as a SPCP. This systematic review will, to our knowledge, be the first to synthesise studies that examine practitioner-reported challenges. We hope that better understanding the ethical challenges experienced by healthcare practitioners working in palliative care in their day-to-day practice will help to inform:

a) Palliative Care Education. This synthesis of the evidence will help identify ethics training needs and inform educational training curricula for all those involved in palliative care provision.
b) Clinical Ethics Education. This review will further develop the evidence base that supports design of more general ethics curricula (e.g. for philosophers, lawyers or social scientists working in or learning bioethics), including revision of the topics included in these curricula and critical examination of the assumptions behind these choices.
c) Research. This work will establish the state of the science in this field and provide a sound basis on which to identify palliative care bioethics research priorities.

The protocol design decisions we have made are associated with a number of potential limitations. First, the search strategy uses methodological filters, which may filter out studies that contain relevant data. Pilot searches were evaluated for study loss using studies known about prior to the review; all were returned by the search strategy. Additional search strategies (hand-searching reference lists and contacting authors of included studies) will also be employed. However, it is possible that a relevant study might not be identified due to mis-classification in the registry or use of another relevant methodology in a novel way.

Secondly, our criteria exclude studies that focus on the ethical challenges of a particular aspect of palliative care, for example the ethical challenges within palliative sedation or advance care planning. Studies that focus on particular aspects of practice are likely to generate granular data about particular challenges. This level of data would allow for better understanding of the complex nature of these topics. However, as Hermsen and Ten Have shown, the topics selected by authors for investigation in this manner may not represent the challenges that are faced in day-to-day practice.[24] We therefore take the opinion that a model of ethical challenges developed from only

For peer review only - http://bmjopen.bmj.com/site/about/guidelines.xhtml

inductive data would form a valuable evidence-based foundation on which to base future in-depth research.

Thirdly, quality assessment of qualitative research is a contested area, with multiple tools available and often poor correlation between methods.[35] The MMAT contains fewer criteria to assess study quality than methodology specific tools, for example the CASP Qualitative Check List.[36] This may lead to an incorrect over or under assessment of a study's inherent bias. However, as we will not exclude studies based on their MMAT scores we feel the ability to directly compare studies of differing methodologies has significant benefits in terms of utility to this review.

Reporting

This study protocol has been designed with reference to Preferred Reporting Items for Systematic Reviews and Meta-Analyses Protocols (PRISMA-P) [30] (see supplementary file 1 for checklist). The review will be reported in line with the Preferred Reporting Items for Systematic Reviews and Meta-Analyses (PRISMA) statement.[37]

INSERT SUPPLEMENTARY FILE 1

Acknowledgements

The authors would like to thank Alison Richards and Sarah Herring for their assistance in developing the search strategies for this review.

Authors Contributions

GS, EB, LES, and RH contributed to the conceptualisation of the review and the protocol. GS and LES wrote the manuscript draft. GS, MD, RH and EB developed the search strategy. All authors revised and edited the draft manuscript and search strategy. All authors approved the manuscript.

Funding Statement

This work was supported by Wellcome Trust Research Award for Health Professionals (208129/Z/17/Z) for GS. The sponsor is the University of Bristol. Neither the funder nor the sponsor had any input into the design of this protocol.

Competing Interests Statement

All authors confirm they have no conflicts of interest.

Supplementary File

Completed Prisma-P Checklist

For peer review only - http://bmjopen.bmj.com/site/about/guidelines.xhtml

REFERENCES

1 World Health Organisation. WHO | WHO Definition of Palliative Care. WHO. http://www.who.int/cancer/palliative/definition/en/ (accessed 20 Aug 2018).

2 Connor SR, Bermedo MCS, Worldwide Palliative Care Alliance, *et al. Global Atlas of Palliative Care at the End of Life*. 2014. http://www.who.int/nmh/Global_Atlas_of_Palliative_Care.pdf (accessed 22 Nov 2018).

3 Connor SR, Downing J, Marston J. Estimating the global need for palliative care for children: A cross-sectional analysis. *J Pain Symptom Manage* Published Online First: 17 October 2016. doi:10.1016/j.jpainsymman.2016.08.020

4 Lynch T, Connor S, Clark D. Mapping levels of palliative care development: a global update. *J Pain Symptom Manage* 2013;**45**:1094–106. doi:10.1016/j.jpainsymman.2012.05.011

5 Knaul FM, Farmer PE, Krakauer EL, *et al.* Alleviating the access abyss in palliative care and pain relief—an imperative of universal health coverage: the Lancet Commission report. *The Lancet* Published Online First: October 2017. doi:10.1016/S0140-6736(17)32513-8

6 Etkind SN, Bone AE, Gomes B, *et al.* How many people will need palliative care in 2040? Past trends, future projections and implications for services. *BMC Med* 2017;**15**:102. doi:10.1186/s12916-017-0860-2

7 Wagner K-H, Brath H. A global view on the development of non communicable diseases. *Prev Med* 2012;**54**:S38–41. doi:10.1016/j.ypmed.2011.11.012

8 Powell RA, Ali Z, Luyirika E, *et al.* Out of the shadows: non-communicable diseases and palliative care in Africa. *BMJ Support Palliat Care* Published Online First: 21 September 2015. doi:10.1136/bmjspcare-2014-000751

9 Hermsen MA, ten Have HAMJ. Practical ethics of palliative care. *Am J Hosp Palliat Med* 2003;**20**:97–8. doi:10.1177/104990910302000206

10 Clinch A, Le B. Withdrawal of mechanical ventilation in the home: A case report and review of the literature. *Palliat Med* 2011;**25**:378–81. doi:10.1177/0269216310396113

11 Guo Q, Jacelon CS. An integrative review of dignity in end-of-life care. *Palliat Med* 2014;**28**:931–40. doi:10.1177/0269216314528399

12 Woods S. Respect for Persons, Autonomy and Palliative Care. *Med Health Care Philos Eur J* 2005;**8**:243–53.

13 Broeckaert B. Palliative Sedation, Physician-Assisted Suicide, and Euthanasia: "Same, Same, but Different"? *Am J Bioeth* 2011;**11**:62–4.

14 ten Have H, Welie JVM. Palliative Sedation Versus Euthanasia: An Ethical Assessment. *J Pain Symptom Manage* 2014;**47**:123–36. doi:10.1016/j.jpainsymman.2013.03.008

15 Joint Royal Colleges of Physicians Training Board. Specialty Training Curriculum for Palliative Medicine 2010 (Ammednments 2014).

For peer review only - http://bmjopen.bmj.com/site/about/guidelines.xhtml

2014.https://www.jrcptb.org.uk/sites/default/files/2010%20Palliative%20medicine%20%28amendments%202014%29.pdf (accessed 16 Aug 2018).

16 Downing J, Boucher S, Marston J. Identification of training needs for children's palliative care. *Palliat Med* Published Online First: June 2012. doi:10.1177/0269216312446391

17 Ives J. A method of reflexive balancing in a pragmatic, interdisciplinary and reflexive bioethics. *Bioethics* 2014;**28**:302–12. doi:10.1111/bioe.12018

18 Singh I. Evidence, Epistemology and Empirical Bioethics. In: Cribb A, Ives J, Dunn M, eds. *Empirical Bioethics: Theoretical and Practical Perspectives*. Cambridge: : Cambridge University Press 2016. 67–83. doi:10.1017/9781139939829.006

19 Ahl H. Motivation in adult education: a problem solver or a euphemism for direction and control? *Int J Lifelong Educ* 2006;**25**:385–405. doi:10.1080/02601370600772384

20 Taylor K, Lamoreaux A. Teaching with the brain in mind. *New Dir Adult Contin Educ* 2008;**2008**:49–59. doi:10.1002/ace.305

21 Ferrell BR, Novy D, Sullivan MD, *et al.* Ethical dilemmas in pain management. *J Pain* 2001;**2**:171–80. doi:10.1054/jpai.2001.21596

22 Braunack-Mayer AJ. What makes a problem an ethical problem? An empirical perspective on the nature of ethical problems in general practice. *J Med Ethics* 2001;**27**:98–103. doi:10.1136/jme.27.2.98

23 Hermsen M, van der Donk M. Nurses' Moral Problems in Dialisys. *Nurs Ethics* 2009;**16**:184–91. doi:10.1177/0969733008100078

24 Hermsen MA, Ten Have HAMJ. Moral Problems in Palliative Care Practice: A Qualitative Study. *Med Health Care Philos Eur J* 2003;**6**:263–72.

25 Hermsen MA, ten Have HA. Moral problems in palliative care journals. *Palliat Med* 2001;**15**:425–431.

26 Guevara-López U, Altamirano-Bustamante MM, Viesca-Treviño C. New frontiers in the future of palliative care: real-world bioethical dilemmas and axiology of clinical practice. *BMC Med Ethics* 2015;**16**. doi:10.1186/s12910-015-0003-2

27 Butler A, Hall H, Copnell B. A Guide to Writing a Qualitative Systematic Review Protocol to Enhance Evidence-Based Practice in Nursing and Health Care: The Qualitative Systematic Review Protocol. *Worldviews Evid Based Nurs* 2016;**13**:241–9. doi:10.1111/wvn.12134

28 ISSG Search Filters Resource. https://sites.google.com/a/york.ac.uk/issg-search-filters-resource/home (accessed 15 Aug 2018).

29 Cook AM, Finlay IG, Edwards AGK, *et al.* Efficiency of Searching the Grey Literature in Palliative Care. *J Pain Symptom Manage* 2001;**22**:797–801. doi:10.1016/S0885-3924(01)00315-3

30 Shamseer L, Moher D, Clarke M, *et al.* Preferred reporting items for systematic review and meta-analysis protocols (PRISMA-P) 2015: elaboration and explanation. *BMJ* 2015;**349**:g7647. doi:10.1136/bmj.g7647

For peer review only - http://bmjopen.bmj.com/site/about/guidelines.xhtml

31 Popay J, Roberts H, Sowden A, *et al.* Guidance on the conduct of narrative synthesis in systematic reviews. *Prod ESRC Methods Programme Version* 2006;**1**:b92.

32 Thomas J, Harden A. Methods for the thematic synthesis of qualitative research in systematic reviews. *BMC Med Res Methodol* 2008;**8**:45. doi:10.1186/1471-2288-8-45

33 Pluye P, Robert E, Cargo M, *et al.* Proposal: A mixed methods appraisal tool for systematic mixed studies reviews. 2011.http://mixedmethodsappraisaltoolpublic.pbworks.com/w/file/fetch/84371689/MMAT%202011%20criteria%20and%20tutorial%202011-06-29updated2014.08.21.pdf (accessed 9 Oct 2018).

34 Lewin S, Booth A, Glenton C, *et al.* Applying GRADE-CERQual to qualitative evidence synthesis findings: introduction to the series. *Implement Sci* 2018;**13**:2. doi:10.1186/s13012-017-0688-3

35 Dixon-Woods M, Sutton A, Shaw R, *et al.* Appraising qualitative research for inclusion in systematic reviews: a quantitative and qualitative comparison of three methods. *J Health Serv Res Policy* 2007;**12**:42–7. doi:10.1258/135581907779497486

36 Critical Appraisal Skills Programme. CASP Qualitative Checklist. 2018.https://casp-uk.net/wp-content/uploads/2018/01/CASP-Qualitative-Checklist.pdf (accessed 16 Aug 2018).

37 Moher D, Liberati A, Tetzlaff J, *et al.* Preferred reporting items for systematic reviews and meta-analyses: the PRISMA statement. *BMJ* 2009;**339**:b2535. doi:10.1136/bmj.b2535

For peer review only

For peer review only - http://bmjopen.bmj.com/site/about/guidelines.xhtml

PRISMA-P (Preferred Reporting Items for Systematic review and Meta-Analysis Protocols) 2015 checklist: recommended items to address in a systematic review protocol*

Section and topic	Item No	Checklist item	Page in Text
**ADMINISTRATIVE INFORMATION**			
Title:			
Identification	1a	Identify the report as a protocol of a systematic review	1
Update	1b	If the protocol is for an update of a previous systematic review, identify as such	n/a
Registration	2	If registered, provide the name of the registry (such as PROSPERO) and registration number	2
Authors:			
Contact	3a	Provide name, institutional affiliation, e-mail address of all protocol authors; provide physical mailing address of corresponding author	1
Contributions	3b	Describe contributions of protocol authors and identify the guarantor of the review	12
Amendments	4	If the protocol represents an amendment of a previously completed or published protocol, identify as such and list changes; otherwise, state plan for documenting important protocol amendments	n/a
Support:			
Sources	5a	Indicate sources of financial or other support for the review	12
Sponsor	5b	Provide name for the review funder and/or sponsor	12
Role of sponsor or funder	5c	Describe roles of funder(s), sponsor(s), and/or institution(s), if any, in developing the protocol	12
**INTRODUCTION**			
Rationale	6	Describe the rationale for the review in the context of what is already known	4,5
Objectives	7	Provide an explicit statement of the question(s) the review will address with reference to participants, interventions, comparators, and outcomes (PICO)	5
**METHODS**			
Eligibility criteria	8	Specify the study characteristics (such as PICO, study design, setting, time frame) and report characteristics (such as years considered, language, publication status) to be used as criteria for eligibility for the review	5,6,7 & table 1
Information sources	9	Describe all intended information sources (such as electronic databases, contact with study authors, trial registers or other grey literature sources) with planned dates of coverage	7,9
Search strategy	10	Present draft of search strategy to be used for at least one electronic database, including planned limits, such that it could be repeated	7 & box 1

For peer review only

Study records:			
Data management	11a	Describe the mechanism(s) that will be used to manage records and data throughout the review	9
Selection process	11b	State the process that will be used for selecting studies (such as two independent reviewers) through each phase of the review (that is, screening, eligibility and inclusion in meta-analysis)	9
Data collection process	11c	Describe planned method of extracting data from reports (such as piloting forms, done independently, in duplicate), any processes for obtaining and confirming data from investigators	9
Data items	12	List and define all variables for which data will be sought (such as PICO items, funding sources), any pre-planned data assumptions and simplifications	9
Outcomes and prioritization	13	List and define all outcomes for which data will be sought, including prioritization of main and additional outcomes, with rationale	6 & table 1
Risk of bias in individual studies	14	Describe anticipated methods for assessing risk of bias of individual studies, including whether this will be done at the outcome or study level, or both; state how this information will be used in data synthesis	10
Data synthesis	15a	Describe criteria under which study data will be quantitatively synthesised	n/a
	15b	If data are appropriate for quantitative synthesis, describe planned summary measures, methods of handling data and methods of combining data from studies, including any planned exploration of consistency (such as I^2, Kendall's τ)	n/a
	15c	Describe any proposed additional analyses (such as sensitivity or subgroup analyses, meta-regression)	n/a
	15d	If quantitative synthesis is not appropriate, describe the type of summary planned	10
Meta-bias(es)	16	Specify any planned assessment of meta-bias(es) (such as publication bias across studies, selective reporting within studies)	n/a
Confidence in cumulative evidence	17	Describe how the strength of the body of evidence will be assessed (such as GRADE)	10

*** It is strongly recommended that this checklist be read in conjunction with the PRISMA-P Explanation and Elaboration (cite when available) for important clarification on the items. Amendments to a review protocol should be tracked and dated. The copyright for PRISMA-P (including checklist) is held by the PRISMA-P Group and is distributed under a Creative Commons Attribution Licence 4.0.**

From: Shamseer L, Moher D, Clarke M, Ghersi D, Liberati A, Petticrew M, Shekelle P, Stewart L, PRISMA-P Group. Preferred reporting items for systematic review and meta-analysis protocols (PRISMA-P) 2015: elaboration and explanation. BMJ. 2015 Jan 2;349(jan02 1):g7647.

For peer review only

BMJ Open

Real-world Ethics in Palliative Care: Protocol for a Systematic Review of the Ethical Challenges Reported by Specialist Palliative Care Practitioners in their Clinical Practice

Journal:	*BMJ Open*
Manuscript ID	bmjopen-2018-028480.R1
Article Type:	Protocol
Date Submitted by the Author:	28-Feb-2019
Complete List of Authors:	Schofield, Guy; University of Bristol, Centre for Ethics in Medicine<br>Brangan, Emer; University Hospitals Bristol NHS Foundation Trust, The National Institute for Health Research Collaboration for Leadership in Applied Health Research and Care West (NIHR CLAHRC West)<br>Dittborn, Mariana; King's College London, Florence Nightingale Faculty of Nursing, Midwifery & Palliative Care<br>Huxtable, Richard; University of Bristol, Centre for Ethics in Medicine<br>Selman, Lucy; University of Bristol School of Social and Community Medicine
**Primary Subject Heading**:	Palliative care
Secondary Subject Heading:	Ethics
Keywords:	PALLIATIVE CARE, Empirical Ethics, MEDICAL ETHICS, Systematic Review, Ethical Challenges

SCHOLARONE™
Manuscripts

Real-world Ethics in Palliative Care: Protocol for a Systematic Review of the Ethical Challenges Reported by Specialist Palliative Care Practitioners in their Clinical Practice

Guy Schofield[1*], Emer Brangan[2], Mariana Dittborn[3], Richard Huxtable[1], Lucy Ellen Selman[4]

[1] Centre for Ethics in Medicine, Population Health Sciences, Bristol Medical School, University of Bristol, Canynge Hall, 39 Whatley Road, Bristol, BS8 2PS, United Kingdom Tel: +44(0)117 33 14550. guy.schofield@bristol.ac.uk Orcid ID: 0000-0002-9055-292X [* Corresponding Author]

[2] The National Institute for Health Research Collaboration for Leadership in Applied Health Research and Care West (NIHR CLAHRC West) at University Hospitals Bristol NHS Foundation Trust, Bristol, UK

[3] MSc Student, Florence Nightingale Faculty of Nursing, Midwifery & Palliative Care, King's College London, London, UK

[4] Population Health Sciences, Medical School, University of Bristol, Bristol, UK

For peer review only

Current word count: 2576

Keywords

Palliative Care, Empirical Ethics, Medical Ethics, Systematic Review, Ethical Challenges

Abstract

Introduction

Ethical issues arise daily in the delivery of palliative care. Despite much (largely theoretical) literature, evidence from specialist palliative care practitioners (SPCPs) about real-world ethical challenges has not previously been synthesised. This evidence is crucial to inform education and training and adequately support staff. The aim of this systematic review is to synthesise the evidence regarding the ethical challenges which SPCPs encounter during clinical practice.

Methods and Analysis

We will conduct a systematic review with narrative synthesis of empirical studies that use inductive methods to describe the ethical challenges reported by SPCPs. We will search multiple databases (MEDLINE, Philosopher's Index, EMBASE, PsycINFO, LILACS, WHOLIS, Web of Science and CINAHL) without time, language or geographical restrictions. Keywords will be developed from scoping searches, consultation with information specialists, and reference to key systematic reviews in palliative care and bioethics. Reference lists of included studies will be hand-searched. 10% of retrieved titles and abstracts will be independently dual screened, as will all full text papers. Quality will be dual assessed using the Mixed-Methods Appraisal Tool (2018). Narrative synthesis following Popay et al (2006) will be used to synthesise findings. The strength of resulting recommendations will be assessed using the Grading of Recommendations Assessment, Development and Evaluation approach for qualitative evidence (GRADE-CERQual).

Ethics and Dissemination

As this review will include only published data, no specific ethical approval is required.

We anticipate that the systematic review will be of interest to palliative care practitioners of all backgrounds, and educators in palliative care and medical ethics. Findings will be presented at conferences and published open access in a peer-reviewed journal.

PROSPERO Registration number: CRD42018105365

For peer review only - http://bmjopen.bmj.com/site/about/guidelines.xhtml

Strengths and Limitations

i) The systematic review search strategy utilises a broad range of electronic databases, including those which index philosophical as well as clinical research and international publications.

ii) This global review benefits from no language, time or location restrictions in the search strategy.

iii) The use of peer-reviewed filters for qualitative and survey-based methodologies may lead to loss of some relevant studies.

iv) The exclusion of studies investigating single ethical issues, such as palliative sedation, risks reducing the depth of detail that will be incorporated into the final synthesis.

v) However, the benefit of including only inductive studies reporting SPCP real-world experiences is that the resultant synthesis will represent only those topics that are directly reported by SPCPs, and therefore better reflect the real-world context of their practice.

For peer review only

For peer review only - http://bmjopen.bmj.com/site/about/guidelines.xhtml

INTRODUCTION

Palliative care is a holistic approach to the care of patients with life-limiting illness that aims to maximise quality of life.[1] The focus of care includes both the patient and those close and important to them, such as their family.

Despite the increasing global provision of palliative care services, the need for palliative care is growing and unmet.[2,3] In 2011, 74% of countries worldwide had either no, or only isolated palliative care services.[4] The 2017 Lancet Commission Report estimated that globally 61.1m people required specialist palliative care input in 2015.[5] The majority of these people live in Low and Middle Income Countries, where provision of specialist palliative care is highly variable; globally, it is estimated that only 14% of those who might benefit from palliative care receive it.[2] In the United Kingdom (UK), modelling predicts that by 2040 there will be both an increase in the absolute numbers of deaths, and, due to multimorbidity and medical complexity, an increase in the percentage of those dying that require specialist palliative care.[6] As the current worldwide epidemic of non-communicable diseases grows, this trend is likely to be replicated.[7,8]

In the theoretical literature, palliative care is frequently connected with moral problems across a wide variety of aspects of clinical care.[9] These include, for example, withdrawing and withholding of interventions,[10] dignity and quality of care,[11] respect for autonomy[12] and palliative sedation.[13,14] However, there is evidence from other areas of healthcare practice that the ethical dilemmas discussed in the literature do not accurately reflect the range of the dilemmas that healthcare workers report experiencing in real-world practice.[15–17] Whilst this phenomenon of a mismatch between lived experience and the academic literature has not previously been systematically examined within palliative care, there is some evidence suggesting it does apply.[18–20] Hermsen and Ten Have,[18] for example, compared the ethical challenges reported by specialist palliative care practitioners (SPCPs) from the Netherlands with those found in the palliative care literature. They found 14 reported ethical challenges with no accompanying literature, and two topics with significant literature (organ donation and engagement with ethical committees), but which were not reported in practice.[18]

We aim to address this knowledge gap by systematically reviewing and synthesising the published evidence regarding the ethical challenges reported by SPCPs, in order to generate an understanding of these real-world challenges. This is crucial to the speciality going forward: the need for training in the ethical aspects of palliative care is recognised as a priority,[21] and a thorough understanding of the ethical context practitioners work within is needed if educators are to generate evidence-based curricula that reflect real world contexts. Education benefits from a robust grounding in the real-

For peer review only - http://bmjopen.bmj.com/site/about/guidelines.xhtml

world experiences of learners: the relevance of educational material is a key factor in adult learner motivation,[22] and processing new material in relation to prior experiences contributes to learning efficiency.[23] Similarly, in the field of bioethics there has recently been an 'empirical turn', central to which is the idea that understanding the real-world context of moral problems is a key part of their analysis.[24] Fundamental to high-quality empirical bioethics is an accurate understanding of context, taking robust empirical evidence as a starting point.[25]

Aim

We aim to systematically review the literature to answer the following research question: what do SPCPs report as ethical challenges that they experience during clinical practice?

METHODS AND ANALYSIS

Eligibility Criteria

This review aims to identify studies that describe the ethical challenges reported by SPCPs in their day-to-day clinical practice. The inclusion and exclusion criteria are summarised in Table 1. Strech et al. describe an adaptation of the population, intervention, comparison, and outcome (PICO) system for systematic reviews that are examining empirical bioethical topics;[26] we use their Methodology, Issue, Participants (MIP) system.

Table 1. Inclusion and exclusion criteria

	Inclusion criteria	Exclusion criteria
Types of participants	Study participants are specialist palliative care practitioners (SPCPs) in a patient care role. We define SPCPs as people working in, or for, a healthcare setting whose main focus is on delivering palliative care (as opposed to clinical contexts where palliative care forms part, but not the main focus, of the care provided).<br><br>This may include (but is not limited to) nurses, doctors, occupational therapist, physiotherapists, dieticians, speech and language therapists, psychologists, other allied health professionals and chaplains.<br><br>Studies with a mixed population where SPCP participants' data are separately presented and can be extracted will be included.	Participants who undertake palliative care tasks as part of their role (e.g. oncologists), but who do not specialise in providing palliative care and do not have palliative care as the main focus of their role.
Context	All geographical settings and all clinical settings where specialist palliative care (SPC) is delivered will be included.	Studies conducted in settings in which SPC is not being delivered.

For peer review only - http://bmjopen.bmj.com/site/about/guidelines.xhtml

For peer review only

Issues	The range of ethical challenges that are reported as experienced by SPCPs during clinical delivery of palliative care.<br><br>The definition of 'ethical challenges' will be intentionally kept broad to capture the maximum number of examples. It includes but is not limited to terms such as ethical issues, moral challenges, moral dilemmas, values, good/bad, right/wrong. Ethical challenges can be labelled as such either by authors or participants.	Studies that utilise survey tools with pre-selected ethical dilemmas that have not been inductively derived based on evidence from SPCPs, and studies that investigate a single aspect of palliative care only will be excluded.<br><br>These study designs are excluded as they proceed from an a priori assumption that their selected issues are relevant. They therefore do not contribute to an inductive exploration of the breadth and type of ethical challenges facing practitioners.
Methodologies	Empirical studies examining, using inductive methods, the ethical challenges reported by SPCPs in their clinical practice. These may include qualitative studies, mixed methods studies (e.g. surveys with free-text responses) or quantitative studies using questionnaires derived inductively through consultation with SPCPs.	Studies not reporting inductively-derived empirical data. These may include studies using questionnaires which include ethical challenges selected a priori, or single-issue studies focussed on an ethical challenge selected a priori by the researchers.
Timeframe	Any time frame up until the search date will be included, contingent on the inception dates of the databases included in the search.	
Type of publications	Peer-reviewed journal publications of empirical research. Papers in any language will be included, with findings translated into English where necessary.	Where no full text is available through the university subscription, study authors will be contacted for full text. If there is no response within two weeks the study will be excluded.<br>The following will also be excluded:<br>- Conference abstracts; however, authors will be contacted for further data/publications.<br>- Editorials, letters, or comment/opinion pieces.<br>- Review articles. Reviews will be used for identification of primary research only.<br>- Book sections.

SPCP = specialist palliative care provider

The review will include peer-reviewed inductive studies in which SPCPs report the ethical challenges they face in their real-world clinical practice, or secondary analyses of such data. Deductive research, in which researchers pre-specify a priori the ethical challenges they focus on, will be excluded. Following Creswell and Plano Clark,[27] deductive research "works from the 'top down', from a theory to hypotheses to data to add to or contradict the theory", while inductive research is "bottom-up, using the participants' views to build broader themes and generate a theory interconnecting the themes" (p. 23).[27] We consider inductive data as that which derives from data collection efforts that occur independently from any attempt to validate a particular theory or hypothesis.

For peer review only - http://bmjopen.bmj.com/site/about/guidelines.xhtml

We will include only inductive studies as we aim to generate a landscape of challenges experienced in the real-world context. Scoping searches identified multiple studies investigating pre-selected ethical challenges within the practice of palliative care. Studies of this type will be excluded as they reflect choices of the study authors rather than the real-world experience of practitioners; including them in the synthesis would risk introducing data that do not reflect SPCPs experiences.

Similarly, studies that explore single ethical challenges in specialist palliative care practice, e.g. palliative sedation or advance care planning, will be excluded. These studies proceed from an a priori assumption that their topic of interest is present in the real-world experience of SPCP's. Excluding them will therefore minimise the risk of introducing ethical challenge data that is not present in the real-world experience of SPCP's

Non-peer reviewed papers, studies not reporting inductive empirical data, book chapters, editorials and theses, case reports, opinion pieces, and reviews will be excluded.

There will be no language or timeframe restrictions.

Search Strategy

Electronic Searches

The following databases, identified in conjunction with subject information specialists and indexing journals containing key papers known to the research team, will be searched: MEDLINE (Ovid interface, 1946 onwards), Philosopher's Index (OVID interface 1940 onwards), EMBASE (OVID interface, 1980 onwards), PsycINFO (OVID interface 1806 onwards), LILACS (http://lilacs.bvsalud.org/en/ 1982 onwards), Web of Science (Clarivate interface, 1900 onwards) and CINAHL (EBSCO interface, 1937 onwards). There will be no language, geographical or time limits. Non-English-language records will be screened by a native speaker of the relevant language. If a non-English-language paper is included in the review it will be translated into English prior to integration in the analysis.

Initial search terms were developed with reference to the key words of major systematic reviews in palliative care and bioethics. Scoping searches suggested that the initial search terms would result in over 20,000 records returned, and that relevant studies would be qualitative (e.g. using interviews or focus groups) or use survey-based methodologies. To increase the specificity of the search, we will therefore apply peer-reviewed methodological filters for these study designs, identified via the InterTASC Information Specialists' Sub-Group Search Filter Resource.[28] The MEDLINE search strategy (see Supplementary File 1) will be checked and modified for the other databases as appropriate.

For peer review only - http://bmjopen.bmj.com/site/about/guidelines.xhtml

INSERT SUPPLEMENTARY FILE 1

Searching Other Resources

Reference lists of included papers will be hand-searched. Corresponding authors of papers meeting the inclusion criteria will be contacted to ascertain if there are other published papers they recommend for review. Authors of conference abstracts will be contacted for peer-reviewed data or follow-up publications if available; both will be included if provided and eligible. Papers that cite the included studies will be screened for inclusion.

A grey literature search will not be conducted. Cook et al. demonstrated that an extensive grey literature search did not benefit the review content of a palliative care systematic review despite the significant resources required to undertake it.[29]

Selection Process

All titles and/or abstracts of retrieved records will be screened to identify papers that potentially meet the inclusion criteria. The first researcher (GS) will screen the full search results. A second researcher (MD) will independently screen a random sample of 10%. Differences in screening between GS and the second reviewer will be discussed with the research team to clarify and refine inclusion/exclusion criteria. Contested papers will be discussed and any that remain unresolved will be examined by third reviewer (LS).

The full text of potentially eligible records will be retrieved and independently assessed for eligibility by two review team members (GS, MD). Any disagreement between them over the eligibility of particular papers will be resolved through discussion with a third reviewer (LS).

Data extraction & management

Search results will be exported and collated in Endnote X8. Records will be de-duplicated and numerical results will be recorded and presented in a flowchart that follows the PRISMA design.[30]

Data extraction will be undertaken independently by two reviewers, using a pre-piloted data extraction form. Disagreements will be resolved through consultation with a third reviewer if necessary. Data items to be extracted from included studies will include: 1) citation details including title, publication year and journal; 2) study setting, methods, participant characteristics, sample size; 3) specified definition/conceptualisation of ethical challenges; 4) key findings, themes and sub-themes; 5) sources of potential bias including funders and evidence of reflexivity. In the event of relevant missing data, corresponding authors will be contacted.

Data Synthesis

For peer review only - http://bmjopen.bmj.com/site/about/guidelines.xhtml

We will undertake a systematic narrative synthesis, following the iterative framework proposed by Popay et al,[31] adapted for a review which does not focus on an intervention: (1) Developing a preliminary synthesis of study findings, (2) Exploring relationships in the data, (3) Assessing the robustness of the synthesis product, and (4) Developing a theoretical model of ethical challenges in the real-world practice of SPCPs. Stage 1 will include integrating the themes and content of qualitative studies; this will be guided by the 'thematic synthesis' approach developed by Thomas and Harden. [32] The narrative synthesis will explore findings within and across included studies, taking into account study quality (see below); identify patterns in the data; and synthesise the described ethical challenges in an overarching framework or model.

Risk of bias (quality) assessment

Scoping searches suggest that multiple study designs may be returned. So that the quality of diverse study designs can be compared, we will use the Mixed-Methods Assessment Tool (MMAT) (2018 Version)[33] which allows for comparison of quality between studies using differing methodologies. We will not use low MMAT scores to exclude studies, but we will reflect on study quality and the effect of lower scoring studies on the resulting synthesis. Two reviewers (GS, MD) will score each of the included studies independently. Any disagreements will be resolved by consulting a third independent reviewer.

While this review is not designed to produce recommendations for clinical practice, it is nevertheless important that we reflect on our confidence in the evidence synthesis. As the focus of the review is on inductively-derived empirical data we will use the GRADE-CERQual framework to do so.[34] CERQual provides a systematic and transparent framework for assessing confidence in individual review findings, based on consideration of four components: (1) methodological limitations, (2) coherence, (3) adequacy of data, and (4) relevance. Assessments of the four components collectively contribute to an overall assessment of whether findings from a qualitative evidence synthesis provide a reasonable representation of the phenomenon of interest.

Ethics and Dissemination

As this review will include only published data, no specific ethical approval is required.

This systematic review will synthesise empirical evidence on the ethical challenges reported by SPCPs. The research team anticipate that it will be of interest to palliative care practitioners of all backgrounds, and educators involved in palliative care or postgraduate ethics training. Findings will be presented at relevant conferences and published in a peer-reviewed journal in open access format.

For peer review only - http://bmjopen.bmj.com/site/about/guidelines.xhtml

Patient and public involvement

Patients and the public were not involved in designing the protocol of this systematic review.

DISCUSSION

Ethical challenges are a significant part of the day-to-day experience of working as a SPCP. This systematic review will, to our knowledge, be the first to synthesise studies that examine practitioner-reported challenges. We hope that better understanding the ethical challenges experienced by healthcare practitioners working in palliative care in their day-to-day practice will help to inform:

a) Palliative Care Education. This synthesis of the evidence will help identify ethics training needs and inform educational training curricula for all those involved in palliative care provision.
b) Clinical Ethics Education. This review will further develop the evidence base that supports design of more general ethics curricula (e.g. for philosophers, lawyers or social scientists working in or learning bioethics), including revision of the topics included in these curricula and critical examination of the assumptions behind these choices.
c) Research. This work will establish the state of the science in this field and provide a sound basis on which to identify palliative care bioethics research priorities.

The protocol design decisions we have made are associated with potential limitations. First, the search strategy uses methodological filters. While this accords with Strech et al's recommendation that empirical bioethics reviews limit the number of methodologies that are included,[26] this approach may filter out studies that contain relevant data. Pilot searches were evaluated for study loss using studies known about prior to the review; all were returned by the search strategy. Additional search strategies (hand-searching reference lists and contacting authors of included studies) will also be employed. However, it is possible that a relevant study might not be identified due to mis-classification in the registry or use of another relevant methodology in a novel way.

Secondly, our criteria exclude studies that are not inductive in nature, to ensure we capture the 'real-world' challenges of clinical practice and mitigate potential bias towards using Western ethical principles as a means of structuring and collecting data on ethical challenges, e.g. in questionnaires. We use authors' descriptions of study design to determine whether the study reported used an inductive or deductive approach. However, even in purely qualitative research, data collection can be structured to varying degrees; this is difficult to determine without access to the raw data used in

For peer review only - http://bmjopen.bmj.com/site/about/guidelines.xhtml

analyses. Notwithstanding this limitation, our inclusion and exclusion criteria are designed to exclude those studies which specifically selected *a priori* which topics were of interest and hence did not allow flexibility in terms of the ethical challenges raised by participants.

Thirdly, we also exclude studies which focus on the ethical challenges of a particular aspect of palliative care, for example the ethical challenges within palliative sedation or advance care planning. Studies that focus on particular aspects of practice are likely to generate granular data about particular challenges. This level of data would allow for better understanding of the complex nature of these topics. However, in their comparison between observed ethical challenges and the content of the palliative care ethics literature, Hermsen and ten Have demonstrate that the topics selected by authors for investigation in this manner may not represent the challenges that are faced in real-world practice.[18] The inclusion of single issue studies would increase the risk of this occurring in this review. To meet our aim of developing a model of ethical challenges based on real-world practice, we will therefore exclude these studies.

Finally, quality assessment of qualitative research is a contested area, with multiple tools available and often poor correlation between methods.[35] The MMAT contains fewer criteria to assess study quality than methodology specific tools, for example the CASP Qualitative Check List.[36] This may lead to an incorrect over- or under-assessment of a study's inherent bias. However, as we will not exclude studies based on their MMAT scores we believe the ability to directly compare studies of differing methodologies has significant benefits in terms of utility to this review.

Reporting

This study protocol has been designed with reference to Preferred Reporting Items for Systematic Reviews and Meta-Analyses Protocols (PRISMA-P)[30] (see supplementary file 2 for checklist). The review will be reported in line with the Preferred Reporting Items for Systematic Reviews and Meta-Analyses (PRISMA) statement.[37]

INSERT SUPPLEMENTARY FILE 2

Acknowledgements

The authors would like to thank Alison Richards and Sarah Herring for their assistance in developing the search strategies for this review, and Huey Yen Chia for their help translating Traditional Chinese language records for screening.

Authors Contributions

For peer review only

GS, LES, EB and RH conceived of the review and developed the protocol. GS, EB, MD and RH developed the search strategy. GS and LES wrote the manuscript draft. All authors revised and edited the draft manuscript and approved the final version.

Funding Statement

This work was supported by Wellcome Trust Research Award for Health Professionals (208129/Z/17/Z) for GS. The sponsor is the University of Bristol. Neither the funder nor the sponsor had any input into the design of this protocol.

Competing Interests Statement

All authors confirm they have no conflicts of interest.

Supplementary Files

Medline Search Strategy

Completed PRISMA-P Checklist

REFERENCES

1 World Health Organisation. WHO | WHO Definition of Palliative Care. WHO. http://www.who.int/cancer/palliative/definition/en/ (accessed 20 Aug 2018).

2 Connor SR, Bermedo MCS, Worldwide Palliative Care Alliance, *et al. Global Atlas of Palliative Care at the End of Life*. 2014. http://www.who.int/nmh/Global_Atlas_of_Palliative_Care.pdf (accessed 22 Nov 2018).

3 Connor SR, Downing J, Marston J. Estimating the global need for palliative care for children: A cross-sectional analysis. *J Pain Symptom Manage* Published Online First: 17 October 2016. doi:10.1016/j.jpainsymman.2016.08.020

4 Lynch T, Connor S, Clark D. Mapping levels of palliative care development: a global update. *J Pain Symptom Manage* 2013;**45**:1094–106. doi:10.1016/j.jpainsymman.2012.05.011

5 Knaul FM, Farmer PE, Krakauer EL, *et al.* Alleviating the access abyss in palliative care and pain relief—an imperative of universal health coverage: the Lancet Commission report. *The Lancet* Published Online First: October 2017. doi:10.1016/S0140-6736(17)32513-8

For peer review only - http://bmjopen.bmj.com/site/about/guidelines.xhtml

6 Etkind SN, Bone AE, Gomes B, *et al.* How many people will need palliative care in 2040? Past trends, future projections and implications for services. *BMC Med* 2017;**15**:102. doi:10.1186/s12916-017-0860-2

7 Wagner K-H, Brath H. A global view on the development of non communicable diseases. *Prev Med* 2012;**54**:S38–41. doi:10.1016/j.ypmed.2011.11.012

8 Powell RA, Ali Z, Luyirika E, *et al.* Out of the shadows: non-communicable diseases and palliative care in Africa. *BMJ Support Palliat Care* Published Online First: 21 September 2015. doi:10.1136/bmjspcare-2014-000751

9 Hermsen MA, ten Have HAMJ. Practical ethics of palliative care. *Am J Hosp Palliat Med* 2003;**20**:97–8. doi:10.1177/104990910302000206

10 Clinch A, Le B. Withdrawal of mechanical ventilation in the home: A case report and review of the literature. *Palliat Med* 2011;**25**:378–81. doi:10.1177/0269216310396113

11 Guo Q, Jacelon CS. An integrative review of dignity in end-of-life care. *Palliat Med* 2014;**28**:931–40. doi:10.1177/0269216314528399

12 Woods S. Respect for Persons, Autonomy and Palliative Care. *Med Health Care Philos Eur J* 2005;**8**:243–53.

13 Broeckaert B. Palliative Sedation, Physician-Assisted Suicide, and Euthanasia: "Same, Same, but Different"? *Am J Bioeth* 2011;**11**:62–4.

14 ten Have H, Welie JVM. Palliative Sedation Versus Euthanasia: An Ethical Assessment. *J Pain Symptom Manage* 2014;**47**:123–36. doi:10.1016/j.jpainsymman.2013.03.008

15 Ferrell BR, Novy D, Sullivan MD, *et al.* Ethical dilemmas in pain management. *J Pain* 2001;**2**:171–80. doi:10.1054/jpai.2001.21596

16 Braunack-Mayer AJ. What makes a problem an ethical problem? An empirical perspective on the nature of ethical problems in general practice. *J Med Ethics* 2001;**27**:98–103. doi:10.1136/jme.27.2.98

17 Hermsen M, van der Donk M. Nurses' Moral Problems in Dialisys. *Nurs Ethics* 2009;**16**:184–91. doi:10.1177/0969733008100078

For peer review only

For peer review only - http://bmjopen.bmj.com/site/about/guidelines.xhtml

18 Hermsen MA, Ten Have HAMJ. Moral Problems in Palliative Care Practice: A Qualitative Study. *Med Health Care Philos Eur J* 2003;**6**:263–72.

19 Hermsen MA, ten Have HA. Moral problems in palliative care journals. *Palliat Med* 2001;**15**:425–431.

20 Guevara-López U, Altamirano-Bustamante MM, Viesca-Treviño C. New frontiers in the future of palliative care: real-world bioethical dilemmas and axiology of clinical practice. *BMC Med Ethics* 2015;**16**. doi:10.1186/s12910-015-0003-2

21 Downing J, Boucher S, Marston J. Identification of training needs for children's palliative care. *Palliat Med* Published Online First: June 2012. doi:10.1177/0269216312446391

22 Ahl H. Motivation in adult education: a problem solver or a euphemism for direction and control? *Int J Lifelong Educ* 2006;**25**:385–405. doi:10.1080/02601370600772384

23 Taylor K, Lamoreaux A. Teaching with the brain in mind. *New Dir Adult Contin Educ* 2008;**2008**:49–59. doi:10.1002/ace.305

24 Ives J. A method of reflexive balancing in a pragmatic, interdisciplinary and reflexive bioethics. *Bioethics* 2014;**28**:302–12. doi:10.1111/bioe.12018

25 Singh I. Evidence, Epistemology and Empirical Bioethics. In: Cribb A, Ives J, Dunn M, eds. *Empirical Bioethics: Theoretical and Practical Perspectives.* Cambridge: : Cambridge University Press 2016. 67–83. doi:10.1017/9781139939829.006

26 Strech D, Synofzik M, Marckmann G. Systematic reviews of empirical bioethics. *J Med Ethics* 2008;**34**:472–7. doi:10.1136/jme.2007.021709

27 Creswell JW, Clark VLP. *Designing and Conducting Mixed Methods Research*. SAGE 2007.

28 ISSG Search Filters Resource. https://sites.google.com/a/york.ac.uk/issg-search-filters-resource/home (accessed 15 Aug 2018).

29 Cook AM, Finlay IG, Edwards AGK, *et al.* Efficiency of Searching the Grey Literature in Palliative Care. *J Pain Symptom Manage* 2001;**22**:797–801. doi:10.1016/S0885-3924(01)00315-3

For peer review only

For peer review only - http://bmjopen.bmj.com/site/about/guidelines.xhtml

30 Shamseer L, Moher D, Clarke M, *et al.* Preferred reporting items for systematic review and meta-analysis protocols (PRISMA-P) 2015: elaboration and explanation. *BMJ* 2015;**349**:g7647. doi:10.1136/bmj.g7647

31 Popay J, Roberts H, Sowden A, *et al.* Guidance on the conduct of narrative synthesis in systematic reviews. *Prod ESRC Methods Programme Version* 2006;**1**:b92.

32 Thomas J, Harden A. Methods for the thematic synthesis of qualitative research in systematic reviews. *BMC Med Res Methodol* 2008;**8**:45. doi:10.1186/1471-2288-8-45

33 Hong Q, Pluye P, Fabregues S, *et al.* Mixed Methods Appraisal Tool (MMAT) Version 2018. 2018.http://mixedmethodsappraisaltoolpublic.pbworks.com/w/file/fetch/127916259/MMAT_2018_criteria-manual_2018-08-01_ENG.pdf (accessed 19 Feb 2019).

34 Lewin S, Booth A, Glenton C, *et al.* Applying GRADE-CERQual to qualitative evidence synthesis findings: introduction to the series. *Implement Sci* 2018;**13**:2. doi:10.1186/s13012-017-0688-3

35 Dixon-Woods M, Sutton A, Shaw R, *et al.* Appraising qualitative research for inclusion in systematic reviews: a quantitative and qualitative comparison of three methods. *J Health Serv Res Policy* 2007;**12**:42–7. doi:10.1258/135581907779497486

36 Critical Appraisal Skills Programme. CASP Qualitative Checklist. 2018.https://casp-uk.net/wp-content/uploads/2018/01/CASP-Qualitative-Checklist.pdf (accessed 16 Aug 2018).

37 Moher D, Liberati A, Tetzlaff J, *et al.* Preferred reporting items for systematic reviews and meta-analyses: the PRISMA statement. *BMJ* 2009;**339**:b2535. doi:10.1136/bmj.b2535

For peer review only

For peer review only - http://bmjopen.bmj.com/site/about/guidelines.xhtml

Medline Search Strategy

1 Ethics/
2 Ethics, Nursing/
3 Ethics, Medical/
4 Ethics, Clinical/
5 exp Ethics, Professional/
6 BIOETHICS/
7 moral*.tw.
8 ethic*.tw.
9 1 or 2 or 3 or 4 or 5 or 6 or 7 or 8
10 Palliative Care/
11 Palliative Medicine/
12 Terminal Care/
13 Hospice Care/
14 Hospices/
15 ((end of life or terminal*) adj3 (ill* or care)).tw.
16 palliat*.tw.
17 hospice*.tw.
18 10 or 11 or 12 or 13 or 14 or 15 or 16 or 17
19 9 and 18
20 exp animals/ not humans/
21 exp Animals, Laboratory/
22 exp Animal Experimentation/
23 exp Models, Animal/
24 (rat or rats or mouse or mice or rodent*).ti.
25 20 or 21 or 22 or 23 or 24
26 19 not 25
27 exp "Surveys and Questionnaires"/
28 survey*.mp.
29 question*.mp.
30 or/27-29
31 ((("semi-structured" or semistructured or unstructured or informal or "in-depth" or indepth or "face-to-face" or structured or guide) adj3 (interview* or discussion* or questionnaire*)) or (focus group* or qualitative or ethnograph* or fieldwork or "field work" or "key informant")).ti,ab. or interviews as topic/ or focus groups/ or narration/ or qualitative research/
32 30 or 31
33 26 and 32

For peer review only

PRISMA-P (Preferred Reporting Items for Systematic review and Meta-Analysis Protocols) 2015 checklist: recommended items to address in a systematic review protocol*

Section and topic	Item No	Checklist item	Page in Text
**ADMINISTRATIVE INFORMATION**			
Title:			
Identification	1a	Identify the report as a protocol of a systematic review	1
Update	1b	If the protocol is for an update of a previous systematic review, identify as such	n/a
Registration	2	If registered, provide the name of the registry (such as PROSPERO) and registration number	2
Authors:			
Contact	3a	Provide name, institutional affiliation, e-mail address of all protocol authors; provide physical mailing address of corresponding author	1
Contributions	3b	Describe contributions of protocol authors and identify the guarantor of the review	11
Amendments	4	If the protocol represents an amendment of a previously completed or published protocol, identify as such and list changes; otherwise, state plan for documenting important protocol amendments	n/a
Support:			
Sources	5a	Indicate sources of financial or other support for the review	12
Sponsor	5b	Provide name for the review funder and/or sponsor	12
Role of sponsor or funder	5c	Describe roles of funder(s), sponsor(s), and/or institution(s), if any, in developing the protocol	12
**INTRODUCTION**			
Rationale	6	Describe the rationale for the review in the context of what is already known	4,5
Objectives	7	Provide an explicit statement of the question(s) the review will address with reference to participants, interventions, comparators, and outcomes (PICO)	5
**METHODS**			
Eligibility criteria	8	Specify the study characteristics (such as PICO, study design, setting, time frame) and report characteristics (such as years considered, language, publication status) to be used as criteria for eligibility for the review	5,6,7 & table 1
Information sources	9	Describe all intended information sources (such as electronic databases, contact with study authors, trial registers or other grey literature sources) with planned dates of coverage	7,8
Search strategy	10	Present draft of search strategy to be used for at least one electronic database, including planned limits, such that it could be repeated	Supplementary file 1

For peer review only

Study records:			
Data management	11a	Describe the mechanism(s) that will be used to manage records and data throughout the review	8
Selection process	11b	State the process that will be used for selecting studies (such as two independent reviewers) through each phase of the review (that is, screening, eligibility and inclusion in meta-analysis)	8
Data collection process	11c	Describe planned method of extracting data from reports (such as piloting forms, done independently, in duplicate), any processes for obtaining and confirming data from investigators	8
Data items	12	List and define all variables for which data will be sought (such as PICO items, funding sources), any pre-planned data assumptions and simplifications	8
Outcomes and prioritization	13	List and define all outcomes for which data will be sought, including prioritization of main and additional outcomes, with rationale	6,7 & table 1
Risk of bias in individual studies	14	Describe anticipated methods for assessing risk of bias of individual studies, including whether this will be done at the outcome or study level, or both; state how this information will be used in data synthesis	9
Data synthesis	15a	Describe criteria under which study data will be quantitatively synthesised	n/a
	15b	If data are appropriate for quantitative synthesis, describe planned summary measures, methods of handling data and methods of combining data from studies, including any planned exploration of consistency (such as I^2, Kendall's τ)	n/a
	15c	Describe any proposed additional analyses (such as sensitivity or subgroup analyses, meta-regression)	n/a
	15d	If quantitative synthesis is not appropriate, describe the type of summary planned	8,9
Meta-bias(es)	16	Specify any planned assessment of meta-bias(es) (such as publication bias across studies, selective reporting within studies)	n/a
Confidence in cumulative evidence	17	Describe how the strength of the body of evidence will be assessed (such as GRADE)	9

*** It is strongly recommended that this checklist be read in conjunction with the PRISMA-P Explanation and Elaboration (cite when available) for important clarification on the items. Amendments to a review protocol should be tracked and dated. The copyright for PRISMA-P (including checklist) is held by the PRISMA-P Group and is distributed under a Creative Commons Attribution Licence 4.0.**

From: Shamseer L, Moher D, Clarke M, Ghersi D, Liberati A, Petticrew M, Shekelle P, Stewart L, PRISMA-P Group. Preferred reporting items for systematic review and meta-analysis protocols (PRISMA-P) 2015: elaboration and explanation. BMJ. 2015 Jan 2;349(jan02 1):g7647.

For peer review only

BMJ Open

Real-world Ethics in Palliative Care: Protocol for a Systematic Review of the Ethical Challenges Reported by Specialist Palliative Care Practitioners in their Clinical Practice

Journal:	*BMJ Open*
Manuscript ID	bmjopen-2018-028480.R2
Article Type:	Protocol
Date Submitted by the Author:	25-Mar-2019
Complete List of Authors:	Schofield, Guy; University of Bristol, Centre for Ethics in Medicine<br>Brangan, Emer; University Hospitals Bristol NHS Foundation Trust, The National Institute for Health Research Collaboration for Leadership in Applied Health Research and Care West (NIHR CLAHRC West)<br>Dittborn, Mariana; King's College London, Florence Nightingale Faculty of Nursing, Midwifery & Palliative Care<br>Huxtable, Richard; University of Bristol, Centre for Ethics in Medicine<br>Selman, Lucy; University of Bristol School of Social and Community Medicine
**Primary Subject Heading**:	Palliative care
Secondary Subject Heading:	Ethics
Keywords:	PALLIATIVE CARE, Empirical Ethics, MEDICAL ETHICS, Systematic Review, Ethical Challenges

SCHOLARONE™
Manuscripts

Real-world Ethics in Palliative Care: Protocol for a Systematic Review of the Ethical Challenges Reported by Specialist Palliative Care Practitioners in their Clinical Practice

Guy Schofield[1*], Emer Brangan[2], Mariana Dittborn[3], Richard Huxtable[1], Lucy Ellen Selman[4]

[1] Centre for Ethics in Medicine, Population Health Sciences, Bristol Medical School, University of Bristol, Canynge Hall, 39 Whatley Road, Bristol, BS8 2PS, United Kingdom Tel: +44(0)117 33 14550. guy.schofield@bristol.ac.uk Orcid ID: 0000-0002-9055-292X [* Corresponding Author]

[2] The National Institute for Health Research Collaboration for Leadership in Applied Health Research and Care West (NIHR CLAHRC West) at University Hospitals Bristol NHS Foundation Trust, Bristol, UK

[3] MSc Student, Florence Nightingale Faculty of Nursing, Midwifery & Palliative Care, King's College London, London, UK

[4] Population Health Sciences, Medical School, University of Bristol, Bristol, UK

For peer review only

Current word count: 2616

Keywords

Palliative Care, Empirical Ethics, Medical Ethics, Systematic Review, Ethical Challenges

Abstract

Introduction

Ethical issues arise daily in the delivery of palliative care. Despite much (largely theoretical) literature, evidence from specialist palliative care practitioners (SPCPs) about real-world ethical challenges has not previously been synthesised. This evidence is crucial to inform education and training and adequately support staff. The aim of this systematic review is to synthesise the evidence regarding the ethical challenges which SPCPs encounter during clinical practice.

Methods and Analysis

We will conduct a systematic review with narrative synthesis of empirical studies that use inductive methods to describe the ethical challenges reported by SPCPs. We will search multiple databases (MEDLINE, Philosopher's Index, EMBASE, PsycINFO, LILACS, WHOLIS, Web of Science and CINAHL) without time, language or geographical restrictions. Keywords will be developed from scoping searches, consultation with information specialists, and reference to key systematic reviews in palliative care and bioethics. Reference lists of included studies will be hand-searched. 10% of retrieved titles and abstracts will be independently dual screened, as will all full text papers. Quality will be dual assessed using the Mixed-Methods Appraisal Tool (2018). Narrative synthesis following Popay et al (2006) will be used to synthesise findings. The strength of resulting recommendations will be assessed using the Grading of Recommendations Assessment, Development and Evaluation approach for qualitative evidence (GRADE-CERQual).

Ethics and Dissemination

As this review will include only published data, no specific ethical approval is required.

We anticipate that the systematic review will be of interest to palliative care practitioners of all backgrounds, and educators in palliative care and medical ethics. Findings will be presented at conferences and published open access in a peer-reviewed journal.

PROSPERO Registration number: CRD42018105365

For peer review only - http://bmjopen.bmj.com/site/about/guidelines.xhtml

Strengths and Limitations

i) The systematic review search strategy utilises a broad range of electronic databases, including those which index philosophical as well as clinical research and international publications.

ii) This global review benefits from no language, time or location restrictions in the search strategy.

iii) The use of peer-reviewed filters for qualitative and survey-based methodologies may lead to loss of some relevant studies.

iv) The exclusion of studies investigating single ethical issues, such as palliative sedation, risks reducing the depth of detail that will be incorporated into the final synthesis.

v) However, the benefit of including only inductive studies reporting SPCP real-world experiences is that the resultant synthesis will represent only those topics that are directly reported by SPCPs, and therefore better reflect the real-world context of their practice.

For peer review only

For peer review only - http://bmjopen.bmj.com/site/about/guidelines.xhtml

INTRODUCTION

Palliative care is a holistic approach to the care of patients with life-limiting illness that aims to maximise quality of life.[1] The focus of care includes both the patient and those close and important to them, such as their family.

Despite the increasing global provision of palliative care services, the need for palliative care is growing and unmet.[2,3] In 2011, 74% of countries worldwide had either no, or only isolated palliative care services.[4] The 2017 Lancet Commission Report estimated that globally 61.1m people required specialist palliative care input in 2015.[5] The majority of these people live in Low and Middle Income Countries, where provision of specialist palliative care is highly variable; globally, it is estimated that only 14% of those who might benefit from palliative care receive it.[2] In the United Kingdom (UK), modelling predicts that by 2040 there will be both an increase in the absolute numbers of deaths, and, due to multimorbidity and medical complexity, an increase in the percentage of those dying that require specialist palliative care.[6] As the current worldwide epidemic of non-communicable diseases grows, this trend is likely to be replicated.[7,8]

In the theoretical literature, palliative care is frequently connected with moral problems across a wide variety of aspects of clinical care.[9] These include, for example, withdrawing and withholding of interventions,[10] dignity and quality of care,[11] respect for autonomy[12] and palliative sedation.[13,14] However, there is evidence from other areas of healthcare practice that the ethical dilemmas discussed in the literature do not accurately reflect the range of the dilemmas that healthcare workers report experiencing in real-world practice.[15–17] Whilst this phenomenon of a mismatch between lived experience and the academic literature has not previously been systematically examined within palliative care, there is some evidence suggesting it does apply.[18–20] Hermsen and Ten Have,[18] for example, compared the ethical challenges reported by specialist palliative care practitioners (SPCPs) from the Netherlands with those found in the palliative care literature. They found 14 reported ethical challenges with no accompanying literature, and two topics with significant literature (organ donation and engagement with ethical committees), but which were not reported in practice.[18]

We aim to address this knowledge gap by systematically reviewing and synthesising the published evidence regarding the ethical challenges reported by SPCPs, in order to generate an understanding of these real-world challenges. This is crucial to the speciality going forward: the need for training in the ethical aspects of palliative care is recognised as a priority,[21] and a thorough understanding of the ethical context practitioners work within is needed if educators are to generate evidence-based curricula that reflect real world contexts. Education benefits from a robust grounding in the real-

For peer review only - http://bmjopen.bmj.com/site/about/guidelines.xhtml

world experiences of learners: the relevance of educational material is a key factor in adult learner motivation,[22] and processing new material in relation to prior experiences contributes to learning efficiency.[23] Similarly, in the field of bioethics there has recently been an 'empirical turn', central to which is the idea that understanding the real-world context of moral problems is a key part of their analysis.[24] Fundamental to high-quality empirical bioethics is an accurate understanding of context, taking robust empirical evidence as a starting point.[25]

Aim

We aim to systematically review the literature to answer the following research question: what do SPCPs report as ethical challenges that they experience during clinical practice?

METHODS AND ANALYSIS

Eligibility Criteria

This review aims to identify studies that describe the ethical challenges reported by SPCPs in their day-to-day clinical practice. The inclusion and exclusion criteria are summarised in Table 1. Strech et al. describe an adaptation of the population, intervention, comparison, and outcome (PICO) system for systematic reviews that are examining empirical bioethical topics;[26] we use their Methodology, Issue, Participants (MIP) system.

Table 1. Inclusion and exclusion criteria

	Inclusion criteria	Exclusion criteria
Types of participants	Study participants are specialist palliative care practitioners (SPCPs) in a patient care role. We define SPCPs as people working in, or for, a healthcare setting whose main focus is on delivering palliative care (as opposed to clinical contexts where palliative care forms part, but not the main focus, of the care provided).<br><br>This may include (but is not limited to) nurses, doctors, occupational therapist, physiotherapists, dieticians, speech and language therapists, psychologists, other allied health professionals and chaplains.<br><br>Studies with a mixed population where SPCP participants' data are separately presented and can be extracted will be included.	Participants who undertake palliative care tasks as part of their role (e.g. oncologists), but who do not specialise in providing palliative care and do not have palliative care as the main focus of their role.
Context	All geographical settings and all clinical settings where specialist palliative care (SPC) is delivered will be included.	Studies conducted in settings in which SPC is not being delivered.

For peer review only - http://bmjopen.bmj.com/site/about/guidelines.xhtml

Issues	The range of ethical challenges that are reported as experienced by SPCPs during clinical delivery of palliative care.<br><br>The definition of 'ethical challenges' will be intentionally kept broad to capture the maximum number of examples. It includes but is not limited to terms such as ethical issues, moral challenges, moral dilemmas, values, good/bad, right/wrong. Ethical challenges can be labelled as such either by authors or participants.	Studies that utilise survey tools with pre-selected ethical dilemmas that have not been inductively derived based on evidence from SPCPs, and studies that investigate a single aspect of palliative care only will be excluded.<br><br>These study designs are excluded as they proceed from an a priori assumption that their selected issues are relevant. They therefore do not contribute to an inductive exploration of the breadth and type of ethical challenges facing practitioners.
Methodologies	Empirical studies examining, using inductive methods, the ethical challenges reported by SPCPs in their clinical practice. These may include qualitative studies, mixed methods studies (e.g. surveys with free-text responses) or quantitative studies using questionnaires derived inductively through consultation with SPCPs.	Studies not reporting inductively-derived empirical data. These may include studies using questionnaires which include ethical challenges selected a priori, or single-issue studies focussed on an ethical challenge selected a priori by the researchers.
Timeframe	Any time frame up until the search date will be included, contingent on the inception dates of the databases included in the search.	
Type of publications	Peer-reviewed journal publications of empirical research. Papers in any language will be included, with findings translated into English where necessary.	Where no full text is available through the university subscription, study authors will be contacted for full text. If there is no response within two weeks the study will be excluded. The following will also be excluded:<br>- Conference abstracts; however, authors will be contacted for further data/publications.<br>- Editorials, letters, or comment/opinion pieces.<br>- Review articles. Reviews will be used for identification of primary research only.<br>- Book sections.

SPCP = specialist palliative care provider

The review will include peer-reviewed studies in which SPCPs report the ethical challenges they face in their real-world clinical practice, or secondary analyses of such data. Studies must derive their data using inductive methods; deductive research, in which researchers pre-specify a priori the ethical challenges they focus on, will be excluded. Following Creswell and Plano Clark,[27] deductive research "works from the 'top down', from a theory to hypotheses to data to add to or contradict the theory", while inductive research is "bottom-up, using the participants' views to build broader themes and generate a theory interconnecting the themes" (p. 23).[27] We consider inductive data as deriving from data collection efforts that are independent from any attempt to validate a particular theory or hypothesis. While much inductive data is qualitative or mixed methods by

For peer review only - http://bmjopen.bmj.com/site/about/guidelines.xhtml

design, it can also include quantitative studies (e.g. surveys using questionnaire items originally derived inductively using qualitative methods rather than specified a priori).

We will include only inductive studies as we aim to generate a landscape of challenges experienced in the real-world context. Scoping searches identified multiple studies investigating pre-selected ethical challenges within the practice of palliative care. Studies of this type will be excluded as they reflect choices of the study authors rather than the real-world experience of practitioners; including them in the synthesis would risk introducing data that do not reflect SPCPs experiences.

Similarly, studies that explore single ethical challenges in specialist palliative care practice, e.g. palliative sedation or advance care planning, will be excluded. These studies proceed from an a priori assumption that their topic of interest is present in the real-world experience of SPCP's. Excluding them will therefore minimise the risk of introducing ethical challenge data that is not present in the real-world experience of SPCP's

Non-peer reviewed papers, studies not reporting inductive empirical data, book chapters, editorials and theses, case reports, opinion pieces, and reviews will be excluded.

There will be no language or timeframe restrictions.

Search Strategy

Electronic Searches

The following databases, identified in conjunction with subject information specialists and indexing journals containing key papers known to the research team, will be searched: MEDLINE (Ovid interface, 1946 onwards), Philosopher's Index (OVID interface 1940 onwards), EMBASE (OVID interface, 1980 onwards), PsycINFO (OVID interface 1806 onwards), LILACS (http://lilacs.bvsalud.org/en/ 1982 onwards), Web of Science (Clarivate interface, 1900 onwards) and CINAHL (EBSCO interface, 1937 onwards). There will be no language, geographical or time limits. Non-English-language records will be screened by a native speaker of the relevant language. If a non-English-language paper is included in the review it will be translated into English prior to integration in the analysis.

Initial search terms were developed with reference to the key words of major systematic reviews in palliative care and bioethics. Scoping searches suggested that the initial search terms would result in over 20,000 records returned, and that relevant studies would be qualitative (e.g. using interviews or focus groups) or use survey-based methodologies. To increase the specificity of the search, we will therefore apply peer-reviewed methodological filters for these study designs, identified via the

For peer review only - http://bmjopen.bmj.com/site/about/guidelines.xhtml

InterTASC Information Specialists' Sub-Group Search Filter Resource.[28] The MEDLINE search strategy (see Supplementary File 1) will be checked and modified for the other databases as appropriate.

INSERT SUPPLEMENTARY FILE 1

Searching Other Resources

Reference lists of included papers will be hand-searched. Corresponding authors of papers meeting the inclusion criteria will be contacted to ascertain if there are other published papers they recommend for review. Authors of conference abstracts will be contacted for peer-reviewed data or follow-up publications if available; both will be included if provided and eligible. Papers that cite the included studies will be screened for inclusion.

A grey literature search will not be conducted. Cook et al. demonstrated that an extensive grey literature search did not benefit the review content of a palliative care systematic review despite the significant resources required to undertake it.[29]

Selection Process

All titles and/or abstracts of retrieved records will be screened to identify papers that potentially meet the inclusion criteria. The first researcher (GS) will screen the full search results. A second researcher (MD) will independently screen a random sample of 10%. Differences in screening between GS and the second reviewer will be discussed with the research team to clarify and refine inclusion/exclusion criteria. Contested papers will be discussed and any that remain unresolved will be examined by third reviewer (LS).

The full text of potentially eligible records will be retrieved and independently assessed for eligibility by two review team members (GS, MD). Any disagreement between them over the eligibility of particular papers will be resolved through discussion with a third reviewer (LS).

Data extraction & management

Search results will be exported and collated in Endnote X8. Records will be de-duplicated and numerical results will be recorded and presented in a flowchart that follows the PRISMA design.[30]

Data extraction will be undertaken independently by two reviewers, using a pre-piloted data extraction form. Disagreements will be resolved through consultation with a third reviewer if necessary. Data items to be extracted from included studies will include: 1) citation details including title, publication year and journal; 2) study setting, methods, participant characteristics, sample size; 3) specified definition/conceptualisation of ethical challenges; 4) key findings, themes and sub-

For peer review only - http://bmjopen.bmj.com/site/about/guidelines.xhtml

themes; 5) sources of potential bias including funders and evidence of reflexivity. In the event of relevant missing data, corresponding authors will be contacted.

Data Synthesis

We will undertake a systematic narrative synthesis, following the iterative framework proposed by Popay et al,[31] adapted for a review which does not focus on an intervention: (1) Developing a preliminary synthesis of study findings, (2) Exploring relationships in the data, (3) Assessing the robustness of the synthesis product, and (4) Developing a theoretical model of ethical challenges in the real-world practice of SPCPs. Stage 1 will include integrating the themes and content of qualitative studies; this will be guided by the 'thematic synthesis' approach developed by Thomas and Harden. [32] The narrative synthesis will explore findings within and across included studies, taking into account study quality (see below); identify patterns in the data; and synthesise the described ethical challenges in an overarching framework or model.

Risk of bias (quality) assessment

Scoping searches suggest that multiple study designs may be returned. So that the quality of diverse study designs can be compared, we will use the Mixed-Methods Assessment Tool (MMAT) (2018 Version)[33] which allows for comparison of quality between studies using differing methodologies. We will not use low MMAT scores to exclude studies, but we will reflect on study quality and the effect of lower scoring studies on the resulting synthesis. Two reviewers (GS, MD) will score each of the included studies independently. Any disagreements will be resolved by consulting a third independent reviewer.

While this review is not designed to produce recommendations for clinical practice, it is nevertheless important that we reflect on our confidence in the evidence synthesis. As the focus of the review is on inductively-derived empirical data we will use the GRADE-CERQual framework to do so.[34] CERQual provides a systematic and transparent framework for assessing confidence in individual review findings, based on consideration of four components: (1) methodological limitations, (2) coherence, (3) adequacy of data, and (4) relevance. Assessments of the four components collectively contribute to an overall assessment of whether findings from a qualitative evidence synthesis provide a reasonable representation of the phenomenon of interest.

Ethics and Dissemination

As this review will include only published data, no specific ethical approval is required.

For peer review only - http://bmjopen.bmj.com/site/about/guidelines.xhtml

This systematic review will synthesise empirical evidence on the ethical challenges reported by SPCPs. The research team anticipate that it will be of interest to palliative care practitioners of all backgrounds, and educators involved in palliative care or postgraduate ethics training. Findings will be presented at relevant conferences and published in a peer-reviewed journal in open access format.

Patient and public involvement

Patients and the public were not involved in designing the protocol of this systematic review.

DISCUSSION

Ethical challenges are a significant part of the day-to-day experience of working as a SPCP. This systematic review will, to our knowledge, be the first to synthesise studies that examine practitioner-reported challenges. We hope that better understanding the ethical challenges experienced by healthcare practitioners working in palliative care in their day-to-day practice will help to inform:

a) Palliative Care Education. This synthesis of the evidence will help identify ethics training needs and inform educational training curricula for all those involved in palliative care provision.
b) Clinical Ethics Education. This review will further develop the evidence base that supports design of more general ethics curricula (e.g. for philosophers, lawyers or social scientists working in or learning bioethics), including revision of the topics included in these curricula and critical examination of the assumptions behind these choices.
c) Research. This work will establish the state of the science in this field and provide a sound basis on which to identify palliative care bioethics research priorities.

The protocol design decisions we have made are associated with potential limitations. First, the search strategy uses methodological filters. While this accords with Strech et al's recommendation that empirical bioethics reviews limit the number of methodologies that are included,[26] this approach may filter out studies that contain relevant data. Pilot searches were evaluated for study loss using studies known about prior to the review; all were returned by the search strategy. Additional search strategies (hand-searching reference lists and contacting authors of included studies) will also be employed. However, it is possible that a relevant study might not be identified due to mis-classification in the registry or use of another relevant methodology in a novel way.

For peer review only - http://bmjopen.bmj.com/site/about/guidelines.xhtml

Secondly, our criteria exclude studies that are not inductive in nature, to ensure we capture the 'real-world' challenges of clinical practice and mitigate potential bias towards using Western ethical principles as a means of structuring and collecting data on ethical challenges, e.g. in questionnaires. We use authors' descriptions of study design to determine whether the study reported used an inductive or deductive approach. However, even in purely qualitative research, data collection can be structured to varying degrees; this is difficult to determine without access to the raw data used in analyses. Notwithstanding this limitation, our inclusion and exclusion criteria are designed to exclude those studies which specifically selected *a priori* which topics were of interest and hence did not allow flexibility in terms of the ethical challenges raised by participants.

Thirdly, we also exclude studies which focus on the ethical challenges of a particular aspect of palliative care, for example the ethical challenges within palliative sedation or advance care planning. Studies that focus on particular aspects of practice are likely to generate granular data about particular challenges. This level of data would allow for better understanding of the complex nature of these topics. However, in their comparison between observed ethical challenges and the content of the palliative care ethics literature, Hermsen and ten Have demonstrate that the topics selected by authors for investigation in this manner may not represent the challenges that are faced in real-world practice.[18] The inclusion of single issue studies would increase the risk of this occurring in this review. To meet our aim of developing a model of ethical challenges based on real-world practice, we will therefore exclude these studies.

Finally, quality assessment of qualitative research is a contested area, with multiple tools available and often poor correlation between methods.[35] The MMAT contains fewer criteria to assess study quality than methodology specific tools, for example the CASP Qualitative Check List.[36] This may lead to an incorrect over- or under-assessment of a study's inherent bias. However, as we will not exclude studies based on their MMAT scores we believe the ability to directly compare studies of differing methodologies has significant benefits in terms of utility to this review.

Reporting

This study protocol has been designed with reference to Preferred Reporting Items for Systematic Reviews and Meta-Analyses Protocols (PRISMA-P)[30] (see supplementary file 2 for checklist). The review will be reported in line with the Preferred Reporting Items for Systematic Reviews and Meta-Analyses (PRISMA) statement.[37]

INSERT SUPPLEMENTARY FILE 2

Acknowledgements

For peer review only - http://bmjopen.bmj.com/site/about/guidelines.xhtml

For peer review only

The authors would like to thank Alison Richards and Sarah Herring for their assistance in developing the search strategies for this review, and Huey Yen Chia for their help translating Traditional Chinese language records for screening.

Authors Contributions

GS, LES, EB and RH conceived of the review and developed the protocol. GS, EB, MD and RH developed the search strategy. GS and LES wrote the manuscript draft. All authors revised and edited the draft manuscript and approved the final version.

Funding Statement

This work was supported by Wellcome Trust Research Award for Health Professionals (208129/Z/17/Z) for GS. The sponsor is the University of Bristol. Neither the funder nor the sponsor had any input into the design of this protocol.

Competing Interests Statement

All authors confirm they have no conflicts of interest.

Supplementary Files

Medline Search Strategy

Completed PRISMA-P Checklist

REFERENCES

1 World Health Organisation. WHO | WHO Definition of Palliative Care. WHO. http://www.who.int/cancer/palliative/definition/en/ (accessed 20 Aug 2018).

2 Connor SR, Bermedo MCS, Worldwide Palliative Care Alliance, *et al. Global Atlas of Palliative Care at the End of Life*. 2014. http://www.who.int/nmh/Global_Atlas_of_Palliative_Care.pdf (accessed 22 Nov 2018).

3 Connor SR, Downing J, Marston J. Estimating the global need for palliative care for children: A cross-sectional analysis. *J Pain Symptom Manage* Published Online First: 17 October 2016. doi:10.1016/j.jpainsymman.2016.08.020

4 Lynch T, Connor S, Clark D. Mapping levels of palliative care development: a global update. *J Pain Symptom Manage* 2013;**45**:1094–106. doi:10.1016/j.jpainsymman.2012.05.011

For peer review only - http://bmjopen.bmj.com/site/about/guidelines.xhtml

5 Knaul FM, Farmer PE, Krakauer EL, *et al.* Alleviating the access abyss in palliative care and pain relief—an imperative of universal health coverage: the Lancet Commission report. *The Lancet* Published Online First: October 2017. doi:10.1016/S0140-6736(17)32513-8

6 Etkind SN, Bone AE, Gomes B, *et al.* How many people will need palliative care in 2040? Past trends, future projections and implications for services. *BMC Med* 2017;**15**:102. doi:10.1186/s12916-017-0860-2

7 Wagner K-H, Brath H. A global view on the development of non communicable diseases. *Prev Med* 2012;**54**:S38–41. doi:10.1016/j.ypmed.2011.11.012

8 Powell RA, Ali Z, Luyirika E, *et al.* Out of the shadows: non-communicable diseases and palliative care in Africa. *BMJ Support Palliat Care* Published Online First: 21 September 2015. doi:10.1136/bmjspcare-2014-000751

9 Hermsen MA, ten Have HAMJ. Practical ethics of palliative care. *Am J Hosp Palliat Med* 2003;**20**:97–8. doi:10.1177/104990910302000206

10 Clinch A, Le B. Withdrawal of mechanical ventilation in the home: A case report and review of the literature. *Palliat Med* 2011;**25**:378–81. doi:10.1177/0269216310396113

11 Guo Q, Jacelon CS. An integrative review of dignity in end-of-life care. *Palliat Med* 2014;**28**:931–40. doi:10.1177/0269216314528399

12 Woods S. Respect for Persons, Autonomy and Palliative Care. *Med Health Care Philos Eur J* 2005;**8**:243–53.

13 Broeckaert B. Palliative Sedation, Physician-Assisted Suicide, and Euthanasia: "Same, Same, but Different"? *Am J Bioeth* 2011;**11**:62–4.

14 ten Have H, Welie JVM. Palliative Sedation Versus Euthanasia: An Ethical Assessment. *J Pain Symptom Manage* 2014;**47**:123–36. doi:10.1016/j.jpainsymman.2013.03.008

15 Ferrell BR, Novy D, Sullivan MD, *et al.* Ethical dilemmas in pain management. *J Pain* 2001;**2**:171–80. doi:10.1054/jpai.2001.21596

16 Braunack-Mayer AJ. What makes a problem an ethical problem? An empirical perspective on the nature of ethical problems in general practice. *J Med Ethics* 2001;**27**:98–103. doi:10.1136/jme.27.2.98

For peer review only - http://bmjopen.bmj.com/site/about/guidelines.xhtml

17 Hermsen M, van der Donk M. Nurses' Moral Problems in Dialisys. *Nurs Ethics* 2009;**16**:184–91. doi:10.1177/0969733008100078

18 Hermsen MA, Ten Have HAMJ. Moral Problems in Palliative Care Practice: A Qualitative Study. *Med Health Care Philos Eur J* 2003;**6**:263–72.

19 Hermsen MA, ten Have HA. Moral problems in palliative care journals. *Palliat Med* 2001;**15**:425–431.

20 Guevara-López U, Altamirano-Bustamante MM, Viesca-Treviño C. New frontiers in the future of palliative care: real-world bioethical dilemmas and axiology of clinical practice. *BMC Med Ethics* 2015;**16**. doi:10.1186/s12910-015-0003-2

21 Downing J, Boucher S, Marston J. Identification of training needs for children's palliative care. *Palliat Med* Published Online First: June 2012. doi:10.1177/0269216312446391

22 Ahl H. Motivation in adult education: a problem solver or a euphemism for direction and control? *Int J Lifelong Educ* 2006;**25**:385–405. doi:10.1080/02601370600772384

23 Taylor K, Lamoreaux A. Teaching with the brain in mind. *New Dir Adult Contin Educ* 2008;**2008**:49–59. doi:10.1002/ace.305

24 Ives J. A method of reflexive balancing in a pragmatic, interdisciplinary and reflexive bioethics. *Bioethics* 2014;**28**:302–12. doi:10.1111/bioe.12018

25 Singh I. Evidence, Epistemology and Empirical Bioethics. In: Cribb A, Ives J, Dunn M, eds. *Empirical Bioethics: Theoretical and Practical Perspectives*. Cambridge: : Cambridge University Press 2016. 67–83. doi:10.1017/9781139939829.006

26 Strech D, Synofzik M, Marckmann G. Systematic reviews of empirical bioethics. *J Med Ethics* 2008;**34**:472–7. doi:10.1136/jme.2007.021709

27 Creswell JW, Clark VLP. *Designing and Conducting Mixed Methods Research*. SAGE 2007.

28 ISSG Search Filters Resource. https://sites.google.com/a/york.ac.uk/issg-search-filters-resource/home (accessed 15 Aug 2018).

29 Cook AM, Finlay IG, Edwards AGK, *et al.* Efficiency of Searching the Grey Literature in Palliative Care. *J Pain Symptom Manage* 2001;**22**:797–801. doi:10.1016/S0885-3924(01)00315-3

For peer review only - http://bmjopen.bmj.com/site/about/guidelines.xhtml

30 Shamseer L, Moher D, Clarke M, *et al.* Preferred reporting items for systematic review and meta-analysis protocols (PRISMA-P) 2015: elaboration and explanation. *BMJ* 2015;**349**:g7647. doi:10.1136/bmj.g7647

31 Popay J, Roberts H, Sowden A, *et al.* Guidance on the conduct of narrative synthesis in systematic reviews. *Prod ESRC Methods Programme Version* 2006;**1**:b92.

32 Thomas J, Harden A. Methods for the thematic synthesis of qualitative research in systematic reviews. *BMC Med Res Methodol* 2008;**8**:45. doi:10.1186/1471-2288-8-45

33 Hong Q, Pluye P, Fabregues S, *et al.* Mixed Methods Appraisal Tool (MMAT) Version 2018. 2018.http://mixedmethodsappraisaltoolpublic.pbworks.com/w/file/fetch/127916259/MMAT_2018_criteria-manual_2018-08-01_ENG.pdf (accessed 19 Feb 2019).

34 Lewin S, Booth A, Glenton C, *et al.* Applying GRADE-CERQual to qualitative evidence synthesis findings: introduction to the series. *Implement Sci* 2018;**13**:2. doi:10.1186/s13012-017-0688-3

35 Dixon-Woods M, Sutton A, Shaw R, *et al.* Appraising qualitative research for inclusion in systematic reviews: a quantitative and qualitative comparison of three methods. *J Health Serv Res Policy* 2007;**12**:42–7. doi:10.1258/135581907779497486

36 Critical Appraisal Skills Programme. CASP Qualitative Checklist. 2018.https://casp-uk.net/wp-content/uploads/2018/01/CASP-Qualitative-Checklist.pdf (accessed 16 Aug 2018).

37 Moher D, Liberati A, Tetzlaff J, *et al.* Preferred reporting items for systematic reviews and meta-analyses: the PRISMA statement. *BMJ* 2009;**339**:b2535. doi:10.1136/bmj.b2535

For peer review only

For peer review only - http://bmjopen.bmj.com/site/about/guidelines.xhtml

Medline Search Strategy

1	Ethics/
2	Ethics, Nursing/
3	Ethics, Medical/
4	Ethics, Clinical/
5	exp Ethics, Professional/
6	BIOETHICS/
7	moral*.tw.
8	ethic*.tw.
9	1 or 2 or 3 or 4 or 5 or 6 or 7 or 8
10	Palliative Care/
11	Palliative Medicine/
12	Terminal Care/
13	Hospice Care/
14	Hospices/
15	((end of life or terminal*) adj3 (ill* or care)).tw.
16	palliat*.tw.
17	hospice*.tw.
18	10 or 11 or 12 or 13 or 14 or 15 or 16 or 17
19	9 and 18
20	exp animals/ not humans/
21	exp Animals, Laboratory/
22	exp Animal Experimentation/
23	exp Models, Animal/
24	(rat or rats or mouse or mice or rodent*).ti.
25	20 or 21 or 22 or 23 or 24
26	19 not 25
27	exp "Surveys and Questionnaires"/
28	survey*.mp.
29	question*.mp.
30	or/27-29
31	((("semi-structured" or semistructured or unstructured or informal or "in-depth" or indepth or "face-to-face" or structured or guide) adj3 (interview* or discussion* or questionnaire*)) or (focus group* or qualitative or ethnograph* or fieldwork or "field work" or "key informant")).ti,ab. or interviews as topic/ or focus groups/ or narration/ or qualitative research/
32	30 or 31
33	26 and 32

For peer review only

PRISMA-P (Preferred Reporting Items for Systematic review and Meta-Analysis Protocols) 2015 checklist: recommended items to address in a systematic review protocol*

Section and topic	Item No	Checklist item	Page in Text
**ADMINISTRATIVE INFORMATION**			
Title:			
Identification	1a	Identify the report as a protocol of a systematic review	1
Update	1b	If the protocol is for an update of a previous systematic review, identify as such	n/a
Registration	2	If registered, provide the name of the registry (such as PROSPERO) and registration number	2
Authors:			
Contact	3a	Provide name, institutional affiliation, e-mail address of all protocol authors; provide physical mailing address of corresponding author	1
Contributions	3b	Describe contributions of protocol authors and identify the guarantor of the review	11
Amendments	4	If the protocol represents an amendment of a previously completed or published protocol, identify as such and list changes; otherwise, state plan for documenting important protocol amendments	n/a
Support:			
Sources	5a	Indicate sources of financial or other support for the review	12
Sponsor	5b	Provide name for the review funder and/or sponsor	12
Role of sponsor or funder	5c	Describe roles of funder(s), sponsor(s), and/or institution(s), if any, in developing the protocol	12
**INTRODUCTION**			
Rationale	6	Describe the rationale for the review in the context of what is already known	4,5
Objectives	7	Provide an explicit statement of the question(s) the review will address with reference to participants, interventions, comparators, and outcomes (PICO)	5
**METHODS**			
Eligibility criteria	8	Specify the study characteristics (such as PICO, study design, setting, time frame) and report characteristics (such as years considered, language, publication status) to be used as criteria for eligibility for the review	5,6,7 & table 1
Information sources	9	Describe all intended information sources (such as electronic databases, contact with study authors, trial registers or other grey literature sources) with planned dates of coverage	7,8
Search strategy	10	Present draft of search strategy to be used for at least one electronic database, including planned limits, such that it could be repeated	Supplementary file 1

For peer review only

Study records:			
Data management	11a	Describe the mechanism(s) that will be used to manage records and data throughout the review	8
Selection process	11b	State the process that will be used for selecting studies (such as two independent reviewers) through each phase of the review (that is, screening, eligibility and inclusion in meta-analysis)	8
Data collection process	11c	Describe planned method of extracting data from reports (such as piloting forms, done independently, in duplicate), any processes for obtaining and confirming data from investigators	8
Data items	12	List and define all variables for which data will be sought (such as PICO items, funding sources), any pre-planned data assumptions and simplifications	8
Outcomes and prioritization	13	List and define all outcomes for which data will be sought, including prioritization of main and additional outcomes, with rationale	6,7 & table 1
Risk of bias in individual studies	14	Describe anticipated methods for assessing risk of bias of individual studies, including whether this will be done at the outcome or study level, or both; state how this information will be used in data synthesis	9
Data synthesis	15a	Describe criteria under which study data will be quantitatively synthesised	n/a
	15b	If data are appropriate for quantitative synthesis, describe planned summary measures, methods of handling data and methods of combining data from studies, including any planned exploration of consistency (such as I^2, Kendall's τ)	n/a
	15c	Describe any proposed additional analyses (such as sensitivity or subgroup analyses, meta-regression)	n/a
	15d	If quantitative synthesis is not appropriate, describe the type of summary planned	8,9
Meta-bias(es)	16	Specify any planned assessment of meta-bias(es) (such as publication bias across studies, selective reporting within studies)	n/a
Confidence in cumulative evidence	17	Describe how the strength of the body of evidence will be assessed (such as GRADE)	9

*** It is strongly recommended that this checklist be read in conjunction with the PRISMA-P Explanation and Elaboration (cite when available) for important clarification on the items. Amendments to a review protocol should be tracked and dated. The copyright for PRISMA-P (including checklist) is held by the PRISMA-P Group and is distributed under a Creative Commons Attribution Licence 4.0.**

From: Shamseer L, Moher D, Clarke M, Ghersi D, Liberati A, Petticrew M, Shekelle P, Stewart L, PRISMA-P Group. Preferred reporting items for systematic review and meta-analysis protocols (PRISMA-P) 2015: elaboration and explanation. BMJ. 2015 Jan 2;349(jan02 1):g7647.

For peer review only
